# Species delimitation and integrative taxonomy of the *Reithrodontomys mexicanus* (Rodentia: Cricetidae) cryptic complex

**DOI:** 10.1002/ece3.10355

**Published:** 2023-07-30

**Authors:** Daily Martínez‐Borrego, Elizabeth Arellano, Francisco X. González‐Cózatl, Sandra M. Ospina‐Garcés, Duke S. Rogers

**Affiliations:** ^1^ Centro de Investigación en Biodiversidad y Conservación Universidad Autónoma del Estado de Morelos Cuernavaca Morelos Mexico; ^2^ Centro de Investigaciones Tropicales Universidad Veracruzana Xalapa Veracruz Mexico; ^3^ Department of Biology and Monte L Bean Life Science Museum Brigham Young University Provo Utah USA

**Keywords:** Cricetidae, cryptic speciation, molecular delimitation, multiples lines of evidence, rodents

## Abstract

Species boundaries are difficult to establish in groups with very similar morphology. As an alternative, it has been suggested to integrate multiple sources of data to clarify taxonomic problems in taxa where cryptic speciation processes have been reported. This is the case of the harvest mouse *Reithrodontomys mexicanus*, which has a problematic taxonomy history as it is considered a complex species. Here, we evaluate the cryptic diversity of *R. mexicanus* using an integrative taxonomy approach in order to detect candidate lineages at the species level. The molecular analysis used one mitochondrial (*cytb*) and two nuclear (*Fgb‐I7* and *IRBP*) genes. Species hypotheses were suggested based on three molecular delimitation methods (mPTP, bGMYC, and STACEY) and *cytb* genetic distance values. Skull and environmental space differences between the delimited species were also tested to complement the discrimination of candidate species. Based on the consensus across the delimitation methods and genetic distance values, four species were proposed, which were mostly supported by morphometric and ecological data: *R. mexicanus* clade I, *R. mexicanus* clade IIA, *R. mexicanus* clade IIIA, and *R. mexicanus* clade IIIB. In addition, the evolutionary relationships between the species that comprise the *R. mexicanus* group were discussed from a phylogenetic approach. Our findings present important taxonomic implications for *Reithrodontomys*, as the number of known species for this genus increases. Furthermore, we highlight the importance of the use of multiple sources of data in systematic studies to establish robust delimitations between species considered taxonomically complex.

## Graphical abstract

We evaluate the cryptic diversity of *Reithrodontomys mexicanus* using an integrative taxonomy approach in order to detect candidate lineages at the species level. Our results suggest at least four candidate species supported by molecular, morphometric, and ecological data, which has important implications for the current taxonomy of the genus.

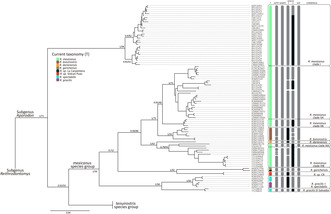

## 1 INTRODUCTION

A continuing challenge in systematics is the determination of which species concept is most appropriate for proposing and delimiting species. Most species have been described using morphological traits (Mayden, 1997), but an increasing number of studies are reporting new species using molecular data (see Jörger & Schrödl, 2013). Controversy around species concepts is largely due to the different nature of the information upon which different concepts are based (de Queiroz, 2007). For example, the Phylogenetic Species Concept (Cracraft, 1989) tends to be used when genetic data are analyzed, while studies focusing on reproductive isolation tend to prefer the Biological Species Concept (Mayr, 1942). Bradley and Baker (2001) proposed delimiting mammal species using the Genetic Species Concept, specifically using genetic distances estimated from the mitochondrial gene Cytochrome b (*cytb*; Baker & Bradley, 2006). In an attempt to eliminate the species problem, de Queiroz (1998, 2005, 2007) proposed the General Lineage Species Concept (GLC). This unified concept uses different species properties, considering elements of the most accepted concepts, and integrates multiple lines of evidence to establish boundaries between species. Therefore, any criteria identifying a separately evolving metapopulation lineage are considered relevant to identify species (de Queiroz, 2005). This multisource approach is known as integrative taxonomy (Dayrat, 2005) and has been widely recommended in systematic studies (see Padial et al., 2010; Sangster, 2018).

Molecular data have been crucial for studying cryptic species in rodents; they allow both the determination of the number of entities comprising a species complex and the delimitation of those entities (D'Elía et al., 2019; and references therein). Using an integrative taxonomy approach, species phylogenetic hypotheses in rodents have been corroborated by other data sources such as morphology, ethology, biogeography, and ecology (e.g., Almendra et al., 2018; Onditi et al., 2021; Rivera et al., 2018). Thus, the congruence among several lines of evidence indicates robust species hypotheses (Dayrat, 2005; Padial et al., 2010).

*Reithrodontomys mexicanus* (Saussure, 1860) is a cricetid rodent with a discontinuous geographic distribution from Mexico to northwestern South America (Hall, 1981; Hooper, 1952). Their populations occupy a variety of habitats, including humid pine‐oak forests, cloud forests, and lowland deciduous forests, and they can generally be found in an altitude range from around 1000 to 3800 m. Recent changes in its taxonomy have been proposed due to divergent lineages detected using different genetic loci and craniodental characteristics (summarized in Martínez‐Borrego et al., 2020). Arellano et al. (2005), employing information from the *cytb* gene, identified three well‐differentiated clades for this species. Based on those findings, they recommended elevating *Reithrodontomys cherrii* to the species level and proposed the existence of an undescribed species distributed in the Sierra Madre Oriental and northern Oaxaca, Mexico (Arellano et al., 2005). Using mitochondrial and nuclear genes, Miller and Engstrom (2008) also suggested that *R. mexicanus* constitutes a cryptic species complex, while Gardner and Carleton (2009) proposed the former *Reithrodontomys m. garichensis* as a species of the *R. mexicanus* group based on craniodental differences compared with the remaining Central American species of the subgenus *Aporodon*.

Thus, as a species complex, *R. mexicanus* sensu lato needs to be reevaluated from a taxonomic point of view. We hypothesize that each of the divergent lineages within *R. mexicanus* constitutes a valid species given their significant genetic, morphological, and ecological differences. Here, our goal is to assess the cryptic diversity of this species based on Arellano et al. (2005) suggestion that it is composed of at least two species. Putative species will be delimited in accordance with the GLC under an integrative taxonomy approach.

## 2 MATERIALS AND METHODS

### 2.1 Molecular analyses of the *Reithrodontomys mexicanus* species complex

#### 2.1.1 Genetic data

**FIGURE 1:**
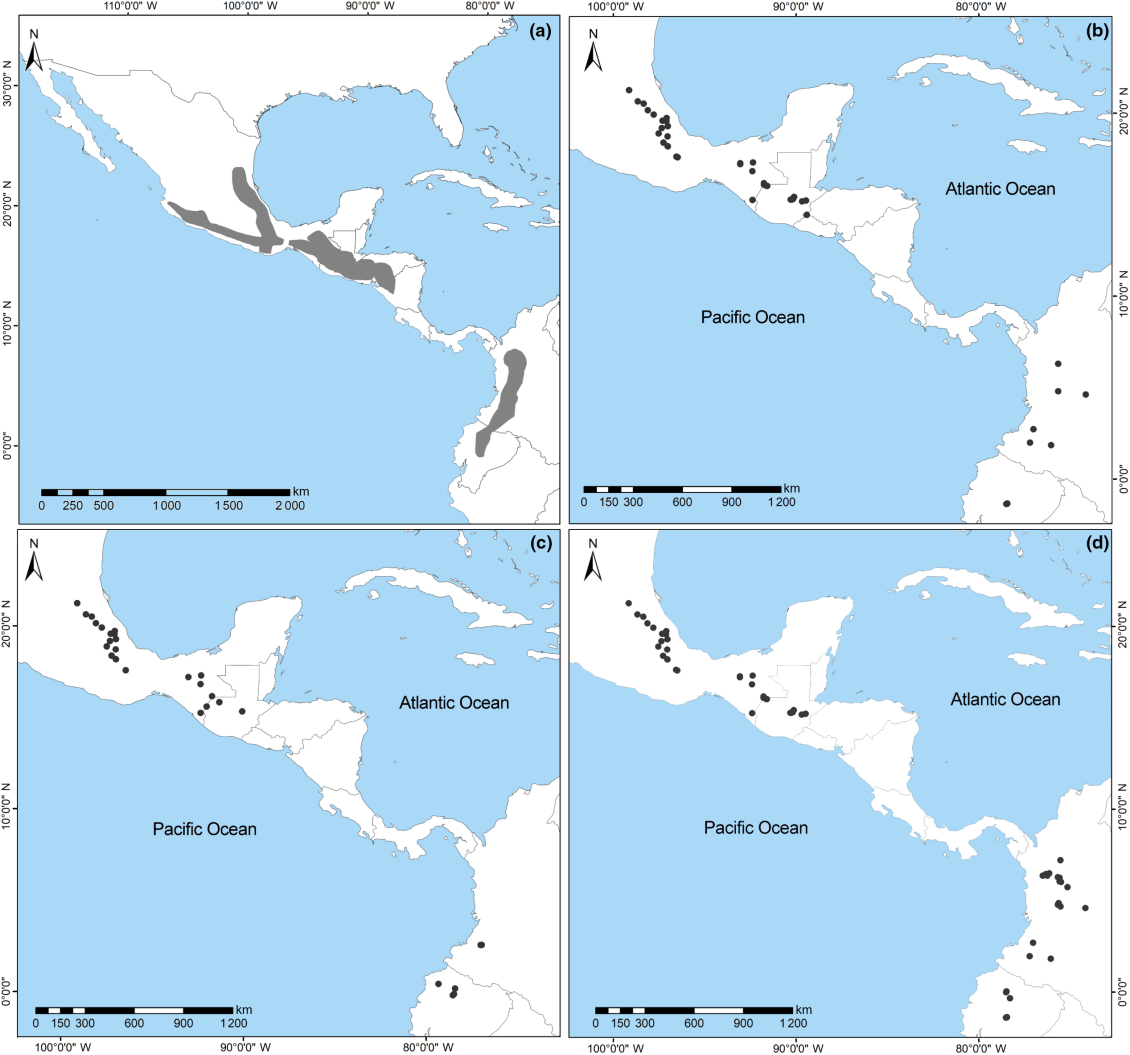
Localities of the *Reithrodontomys mexicanus* specimens used in this study. (a) Current distribution (gray shading) of *R. mexicanus* sensu lato modified from Hooper (1952) and Hall (1981); (b–d) localities utilized in the molecular, geometric morphometric, and ecological analyses, respectively.

DNA sequences from the mitochondrial gene *cytb* and the nuclear genes Intron 7 of the beta fibrinogen (*Fgb‐I7*) and Interphotoreceptor retinoid‐binding protein (*IRBP*) were obtained from collected specimens and tissue loans from mammal collections (Figure 1). Wild‐caught specimens followed the Guidelines approved by the American Society of Mammologists (Sikes & The Animal Care And Use Committee Of The American Society Of Mammalogists, 2016). Genomic DNA extraction, primer information, and PCR procedures for the *cytb* and *Fgb‐I7* genes, as well as sequencing protocols for the amplified PCR products of each gene, are provided in Martínez‐Borrego, Arellano, González‐Cózatl, et al. (2022). Approximately 1140 pb of *cytb* were amplified in 47 individuals of *R. mexicanus*, whereas a 608 bp fragment of *Fgb‐I7* was amplified in 58 individuals. For the *IRBP* gene, the amplification protocol was only successful in three individuals using primers A1 (Stanhope et al., 1992) and B2 (Wickliffe et al., 2003) and the PCR conditions described in Almendra et al. (2018). In individuals for whom other tissues were unavailable, a skin DNA extraction protocol was implemented with modifications from Rogers et al. (2011). Skin DNA amplification was performed using Illustra PuReTaq Ready‐To‐Go PCR Beads and a series of primer pairs as follows: L14724 (Irwin et al., 1991) and H15149 (Kocher et al., 1989) for *cytb*; B17 and Bfib (Wickliffe et al., 2003) for *Fgb‐I7*.

Sequences were edited using Codon Code Aligner v.8.0.2 (CodonCode Corporation) and aligned against a reference in UGENE v.1.32.0 (Okonechnikov et al., 2012) using the MUSCLE method. Eleven species of the genus *Reithrodontomys* were employed as outgroups, representing the distinct species groups defined by Hooper (1952): 5 from the *R. mexicanus* group, 3 from the *R. tenuirostris* group, 2 from the *R. megalotis* group, and 1 from the *R. fulvescens* group. The final data set comprised 109 sequences for *cytb*, 68 for *Fgb‐I7*, and 26 for *IRBP*. The concatenated data set was only available for *cytb* + *Fgb‐I7* (68 sequences, 1750 bp long) and *cytb* + *IRBP* (24, 2064 bp long) because very few individuals presented genetic information for all loci. The information on the sequences generated in this study and those downloaded from GenBank is included in Appendix S1.

#### 2.1.2 Phylogenetic analysis and species delimitation

The evolutionary model and partition scheme (in the case of coding genes) that best fit each data set were estimated in ModelFinder (Kalyaanamoorthy et al., 2017) according to the Bayesian Informative Criterion (Table 1). Maximum Likelihood (ML) and Bayesian Inference (BI) reconstruction methods were used to estimate phylogenetic relationships for each gene and the concatenated data sets. ML analyses were run in IQ‐Tree (Nguyen et al., 2015) using 10,000 Ultrafast Bootstrap replicates (UFBoot; Minh et al., 2013) and the GENESITE resampling strategy to estimate branch support. The BI analyses were run in MrBayes v3.2.6 (Ronquist & Huelsenbeck, 2003) using eight chains in two independent runs (10 million generations each, sampling once every 1000 generations). We used Tracer v1.7.1 (Rambaut et al., 2018) to verify the convergence and seasonality of each run. The posterior probability (pP) was obtained for individual nodes by constructing a majority‐rule consensus after discarding the trees prior to the stationarity phase (10%) as burn‐in. These analyses were implemented within the CIPRES Science Gateway portal (Miller et al., 2012).

**TABLE 1:** Partition schemes and evolutionary models used in the phylogenetic analyses of the *Reithrodontomys mexicanus* species complex.

Gene	Scheme partition	Codon position 1	Codon position 2	Codon position 3
*cytb*	Partitioned (1 + 2 + 3)	TN + I + G4	HKY + F + I + G4	TN + F + G4
*Fgb‐I7*	Noncoding	HKY + F + I		
*IRBP*	Partitioned (1−3 + 2)	TN + F + G4	K2P	

We performed three molecular species delimitation methods: multi‐rate Poisson Tree Processes (mPTP; Kapli et al., 2017), Bayesian General Mixed Yule‐Coalescent Model (bGMYC; Reid & Carstens, 2012), and Species Tree and Classification Estimation, Yarely (STACEY, Jones, 2017). These methods were selected because they do not require a priori assignment of individuals to groups. This allowed us to define cryptic lineages within *R. mexicanus* sensu lato without assumptions regarding their phylogenetic relationships.

The *cytb* gene was used for the two single‐locus methods mPTP and bGMYC. The noncoalescent mPTP method was implemented in the Exelisis Lab platform (http://www.exelixis‐lab.org) using the BI tree and default parameters. The bGMYC coalescent method was implemented in the bGMYC package (Reid & Carstens, 2012) of the R library (R Development Core Team, 2018) using the time‐calibrated tree obtained in BEAST2 and the following input parameters: mcmc = 100,000, burn‐in = 90,000, thinning = 100, t1 = 11, t2 = 16 (based on the upper range of suggested species with mPTP, considering the outgroup), py1 = 0.5, py2 = 1.5, pc1 = 0.1, pc2 = 0.5, start = c (1.0, 0.1, 11), scale = c (20, 10, 5.00). The multi‐locus STACEY method was run using each of the combined datasets (*cytb* + *Fgb‐I7* and *cytb* + *IRBP*). This coalescent method is implemented as a package within BEAST2 and uses a modified birth–death‐collapsed model for the species tree. The species.tree file generated by STACEY was used as input in the SpeciesDelimitationAnalyzer program (speciesDA.jar, www.indriid.com; burn‐in = 1000 collapse height = 0.0001, and similarity cutoff = 1.0) to summarize the posterior tree distribution and calculate the frequency with which each pair of taxa were assigned to the same clade.

The criterion used to define species limits was congruence among the greatest number of delimitation methods, which supports the correct recognition of putative species (Carstens et al., 2013). Genetic distances between lineages proposed as species were estimated using the *cytb* gene in MEGAX (Kumar et al., 2018) under the Kimura two‐parameter evolutionary model (K2P). The 5% *cytb* distance value associated with mammalian sister species recognition (Bradley & Baker, 2001) was set as the genetic distance threshold.

#### 2.1.3 Estimation of divergence time for *cytb* gene

Divergence times between *Aporodon* clades were inferred in BEAST2 (Bouckaert et al., 2014). We obtained a time‐calibrated phylogeny using a birth–death model tree prior and an uncorrelated relaxed lognormal clock. Substitution rate and calibration nodes were established in the same way as in Martínez‐Borrego, Arellano, González‐Cózatl, et al. (2022). MCMC analyses were executed with two runs of 10 million generations each, sampling once every 1000 generations. The BEAST log file was examined in Tracer (Rambaut et al., 2018) to assess the convergence of the independent runs as well as the effective sample size (ESS ≥ 200). A Maxime clade credibility tree was obtained in TreeAnotator after discarding the trees prior to the stationary phase as burn‐in.

### 2.2 Geometric morphometrics of the *Reithrodontomys mexicanus* species complex

#### 2.2.1 Morphometric data

**FIGURE 2:**
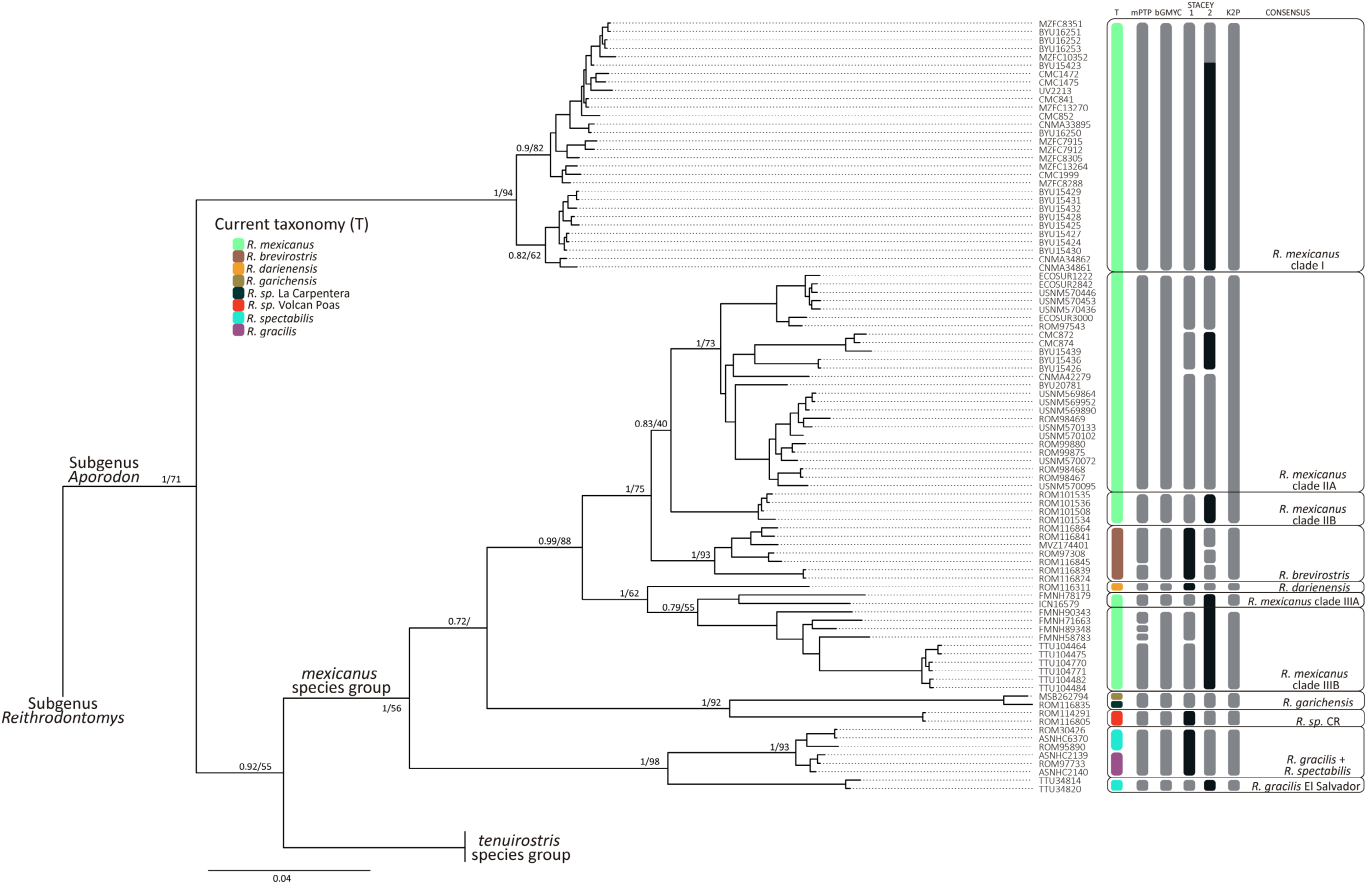
Phylogenetic relationships among species of the *Reithrodontomys mexicanus* group using sequences data of the mitochondrial gene Cytochrome b and the BI reconstruction method. Values on branches represent nodal support for BI/ML analysis, respectively. T = Current taxonomy represented by color bars. Gray bars = identified groups at the species level by single‐locus methods (mPTP/bGMYC = *cytb*) and multiple‐loci method (STACEY 1 = *cytb* + *Fgb‐ I7*; STACEY 2 = *cytb* + *IRBP*) with probability values above 0.95. K2P = identified groups at the species level using Kimura 2‐parameter genetic distances. Consensus = candidate species supported by most of the approaches. When DNA information was not available, a black bar was used. Terminal labels correspond to mammal collection voucher numbers (see Appendix S1).

We examined 335 adult specimens (fully erupted M^3^; following age classes from Arellano et al., 2012) deposited in mammal collections and classified at the time of collection as *R. mexicanus*. However, due to the cryptic diversity reported for this species, only individuals that (1) had a known genetic identity or (2) were collected within less than 60 km of an individual with a known genetic identity were used in the morphometric analyses. In total, 69 specimens (31 males, 38 females) were selected for morphometric analysis (Figure 1 and Appendix S1) and grouped according to the species proposed by the molecular delimitation methods. Individuals from El Salvador and Colombia (*R. mexicanus* clade IIB and clade IIIA in Figure 2) were excluded due to the low sample size (*n* ≤ 5). Digital images of the dorsal and ventral view of the skull of each specimen were taken using an Olympus DP73 Digital Camera and a millimeter rule as a scale bar.

For both views, we digitized landmark and semi‐landmark configurations (Appendix S2) using TPSdig 2.31 (Rohlf, 2015) and assuming positional homology between individuals (Zelditch et al., 2004). Configurations were alignment, rotated, and scaled under a Generalized Procrustes Analysis (Rohlf & Slice, 1990) in the R package geomorph 4.0.4 (Adams et al., 2022). In the case of semi‐landmarks, they were aligned by sliding points along their tangent vectors until reaching the point of minimum bending energy (Bookstein, 1997; Zelditch et al., 2004). Configurations superimposition outputs were the shape variables (Procrustes Distances and Procrustes Coordinates) and the centroid size (CS).

#### 2.2.2 Morphometric comparison between delimited species

We first explored the shape variation in both views of the skull using a principal component analysis of the Procrustes Coordinates to visualize clustering and reduce the dimensionality of the data. We tested skull shape differences related to sex and delimitated species using a Procrustes ANOVA model (Klingenberg & McIntyre, 1998), where we used the Procrustes distance variance to estimate shape variance in each factor. The factorial design included shape as the dependent variable, sex and species as the main factor, and CS as a covariate to also evaluate the effect of the skull size on the shape variation (allometry). Additionally, we used a residual resampling procedure, on the reduced model (Shape~CS), based on 1000 iterations, to assign the significances of the *F* statistic of the model. These analyses were performed in the package geomorph 4.0.4. We also performed pairwise comparison tests to quantify differences in skull shape between delimited species in the R package MORPHO 2.4 (Schlager, 2016) employing the Procrustes Distances, 1000 permutations, and a significance level of *p* ≤ .05. Differences in the CS between sexes and delimitated species were assessed in the R package RRPP (Collyer & Adams, 2018, 2019) using an OLS estimation method and the same resampling procedure described above.

To evaluate cranial shape differences between putative species included in the morphometric analyses, we performed a canonical variates analysis (CVA). Because CVA cannot be performed when the number of variables is greater than the sample size per group (Kovarovic et al., 2011), we only used the shape information from the first 10 principal components (90% and 92% of the variance explained for dorsal and ventral views, respectively). Finally, we performed linear discriminant analysis (LDA) to assess the discrimination of individuals against the different delimited species based on skull shape. A cross‐validation procedure from the CVA scores was employed to obtain the correct discrimination rate, which allowed us to verify the effectiveness of the LDA to discriminate taxa into their correct groups. For these analyses, we used the R packages geomorph and MASS 7.3‐51.4 (Ripley et al., 2013).

### 2.3 Ecological analyses of the *Reithrodontomys mexicanus* species complex

#### 2.3.1 Environmental data

We used occurrence data of the individuals grouped in the delimited species to perform an ecological analysis. Localities from museum databases (not included in the phylogeny) collected within less than 60 km of an individual with a known genetic identity were also included in the ecological analyses (Figure 1; Appendix S1). To reduce georeferencing errors, each geographic coordinate was rectified against the known distribution for *R. mexicanus* sensu lato (Hall, 1981; Hooper, 1952). We removed duplicate point records and thinned the data set by setting a distance between localities ≤3 km using the R package spThing (Aiello–Lammens et al., 2015). This step was required to reduce spatial autocorrelation and avoid overfitting the model derived from the presence of multiple records in the same 1‐km^2^ pixel. A total of 56 localities were employed in the ecological analysis to test niche differences among delimited species. Individuals from El Salvador (clade IIB; see Section 3) could not be included because they were collected from a single locality.

Six bioclimatic variables at 1‐km^2^ resolution were downloaded from Wordclim 2.0 (Fick & Hijmans, 2017; http://www.worldclim.org ): Bio1 = Annual Mean Temperature, Bio5 = Max Temperature of Warmest Month, Bio6 = Min Temperature of Coldest Month, Bio12 = Annual Precipitation, Bio13 = Precipitation of Wettest Month, and Bio14 = Precipitation of Driest Month. These bioclimatic variables were selected based on previous studies that highlighted their importance in ecological studies of small mammals (e.g., Guevara et al., 2018; Martínez‐Borrego, Arellano, Cruz, et al., 2022; Santos et al., 2017; Stanchak & Santana, 2018). Due to the arboreal preferences reported for species of subgenus *Aporodon* (González–Cozátl & Arellano, 2015; Hooper, 1952), we included in our analyses the variable Vegetation Continuous Field (VCF; Hansen et al., 2002; www.landcover.org ) as a measure of the percentage of vegetation cover. This product was obtained from the MODIS sensor using 24 scenarios corresponding to the year 2020, with an original resolution of 250 m. We rescaled the VCF product to the same resolution as the bioclimatic variables using ArGis 10.8 (ESRI, 2020).

#### 2.3.2 Ecological niche modeling (ENM)

We used MaxEnt (Phillips et al., 2006) to build ENMs for each delimited species using the occurrence points and the environmental variables. To improve the models' fit and predictive ability, different parameter settings were evaluated (Muscarella et al., 2014; Warren & Seifert, 2011) using a “checkerboard” method to partition the training and test data. Different values of the regularization multiplier (from 0.5 to 6, in increments of 0.5) and five combinations of feature classes (L = linear, Q = quadratic, H = hinge, P = product, LQHP) were tested. The optimal combination of parameters for each ENM was estimated in the R package ENMeval (Muscarella et al., 2014) and selected according to the lowest delta AICc (Akaike Information Criterion) score. To obtain the ENMs, calibration areas were defined using the minimum convex polygon. Final models were constructed with 50 replicates, and continuous maps were visualized using the cloglog output. The ENMs were binarized using the 10th‐percentile cutoff threshold in ArGis 10.8. Each model's performance was evaluated using the Partial Roc metric implemented in the Niche Toolbox site (http://shiny.conabio.gob.mx:3838/nichetoolb2/), with 1000 bootstrap iterations and *E* = 0.05.

#### 2.3.3 Ecological niche comparison between delimited species

We extracted the values of the environmental variables from each occurrence point in order to determine whether the delimited species differed with respect to the ecological space they occupied. In addition, we performed a CVA to evaluate whether the ecological niche of the delimited species allows them to be segregated based on their environmental characteristics. Multivariate statistical analyses and CVA were run in the R software.

## 3 RESULTS

### 3.1 Cytochrome b phylogeny and molecular species delimitation

The *cytb* gene trees produced identical topologies using both the BI and ML reconstruction methods (Figure 2). The *Aporodon* major clade recovered the *R. mexicanus* and *R. tenuirostris* species groups. Specimens from Costa Rica identified as *R*. sp. by Miller and Engstrom (2008) were recovered as closely related to *R. garichensis*. *Reithrodontomys mexicanus* specimens split into three clades that were well supported in the BI method but weakly supported by ML. Clade I included individuals with geographical distribution in the Sierra Madre Oriental and northern Oaxaca, Mexico. This clade was sister to the *R. mexicanus* and *R. tenuirostris* species groups (pP = 1). Clade II corresponded to specimens distributed in Mexico, Guatemala, and El Salvador and was recovered as the sister group to *brevirostris brevirostris* from Costa Rica (pP = 1). The remaining individuals of *R. mexicanus* were grouped into a clade III with distribution in Colombia and Ecuador and sister to *R. darienensis* from Panama (pP = 1).

The results of the species delimitation methods were not congruent with each other (Figure 2). For the *R. mexicanus* group, the mPTP method identified 15 putative species while 11 were delimited by the bGMYC. The multi‐locus method STACEY suggested 10 species (excluding taxa that were not successfully sequenced), although the proposed species were not the same between data sets (*cytb* + *Fgb‐I7* and *cytb* + *IRBP*). The three methods supported the recognition of *Reithrodontomys darienensis*, *R*. sp. from Volcan Poas, Alajuela, Costa Rica, and *R. garichensis* (including the *R*. sp. individual from La Carpentera, Cartago, Costa Rica), as species. In addition, *Reithrodontomys gracilis* from Yucatán and *Reithrodontomys spectabilis* were considered the same species, excluding specimens of *R. gracilis* distributed in El Salvador, which were delimited as a distinct species. For *R. brevirostris*, each delimitation method proposed different numbers of possible species (from 1 to 3), although bGMYC and K2P genetic distances were congruent in delimiting only one species.

Within the *R. mexicanus* species complex, the *R. mexicanus* clade I was supported at the species level by all three delimitation methods. Within the *R. mexicanus* clade II, STACEY (*cytb* + *Fgb‐I7*) demarcated four possible species, while the other methods delimited only two. Following the recognition of two species, one of them is formed of samples from Mexico and Guatemala (*R. mexicanus* clade IIA), whereas the other corresponded to individuals from El Salvador, which were considered a distinct species under all the methods (*R. mexicanus* clade IIB). Within clade III, the mPTP identified 5 species, whereas bGMYC 2, and STACEY 3 (*cytb* + *Fgb‐I7*). In this clade, the consensus (bGMYC + genetic distances) demarcated two putative species: *R. mexicanus* clade IIIA and *R. mexicanus* clade IIIB. The *cytb* genetic distance values between the species delimitation consensus ranged from 4.96 to 18.18, with the highest value generally between *R. mexicanus* clade I and the other identified species. The *cytb* genetic distance values between delimited taxa in the *R. mexicanus* species group are shown in Table 2 and Appendix S3.

**TABLE 2:** Matrix of Kimura 2‐parameter genetic distances for Cytochrome b gene sequences between recognized and candidate species of the *Reithrodontomys mexicanus* group.

Species delimitation	1	2	3	4	5	6	7	8	9	10
*R. mexicanus* clade I										
*R. mexicanus* clade IIA	15.92									
*R. mexicanus* IIB	15.92	4.96								
*R. mexicanus* clade IIIA	16.86	8.26	8.72							
*R. mexicanus* clade IIIB	15.88	8.32	8.96	6.06						
*R. brevirostris*	16.15	5.68	5.23	8.31	8.34					
*R. darienensis*	16.68	8.48	7.69	8.30	7.54	9.22				
*R. garichensis*	18.18	14.59	12.92	16.09	14.24	14.0	15.62			
*R*. sp. Volcan Poas	17.12	13.83	13.98	14.86	13.93	13.37	13.95	11.31		
*R. gracilis* + *R. spectabilis*	14.70	13.43	13.26	15.53	14.56	12.91	13.86	16.60	15.23	
*R. gracilis* El Salvador	15.16	13.43	14.50	16.72	16.22	14.29	15.55	17.38	16.38	7.91

*Note*: Taxon labels correspond to the delimited groups at the species level by the consensus of the mPTP, bGMYC, and STACEY methods, shown in Figure 2.

### 3.2 Cytochrome b divergence times

**FIGURE 3:**
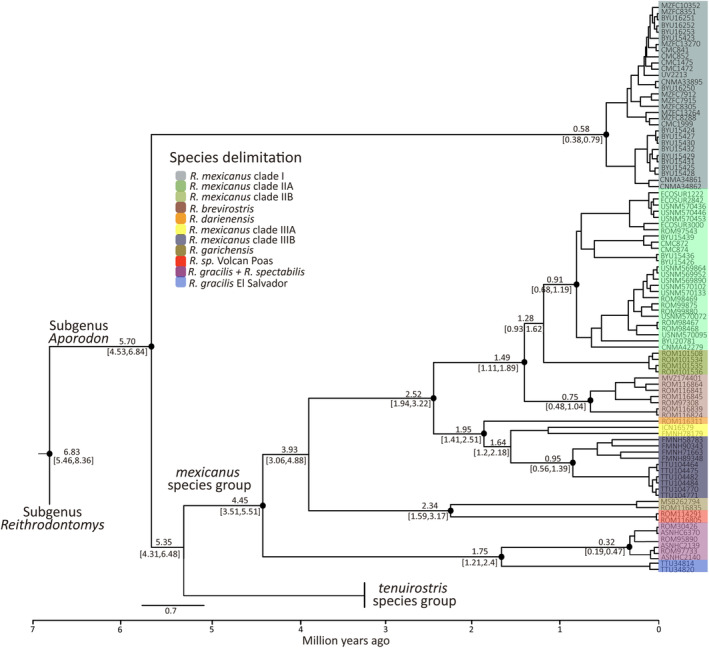
Maximum clade credibility tree obtained with BEAST2 for species of the *Reithrodontomys mexicanus* group using Cytochrome b sequence data. Values above branches represent mean divergence times and below branches are the 95% highest posterior density (HPD) intervals. Black points on nodes represent posterior probability = 1. Terminal labels correspond to mammal collection voucher numbers (see Appendix S1).

The time‐calibrate *cytb* tree (Figure 3) showed that the genus *Reithrodontomys* began to diverge ca. 6.83 mya (95% HPD = 5.46–8.36). The most common recent ancestor of the *Aporodon* clade was at ca. 5.70 mya (95% HPD = 4.53–6.84), when the *R. mexicanus* clade I diverged from the rest of the species of this subgenus. Deep divergences were mostly well supported (pP = 1), except for the split between the *R. mexicanus* and *R. tenuirostris* species groups ca. 5.35 mya (95% HPD = 4.31–6.48) and the split between *R. mexicanus* clade II + *R. brevirostris* + *R. darienensis* + *R. mexicanus* clade III and *R. garichensis* + *R*. sp. Volcan Poas (ca. 3.93, 95% HPD = 3.06–4.88). The earliest divergence in the *R. mexicanus* species group occurred ca. 4.45 mya (95% HPD = 3.51–5.51) when *R. spectabilis* and *R. gracilis* separated from the rest of the species while the most recent divergences occurred between *R. spectabilis* and *R. gracilis* from Yucatan (ca. 0.31 mya, 95% HPD = 0.18–0.48; see Section 4).

### 3.3 Nuclear and concatenated phylogenies

Topologies resulting from the *Fgb‐I7* and *IRBP* nuclear genes were inconsistent with each other and with the *cytb* tree, showing low support values for the ML and BI reconstruction methods. Neither of the nuclear trees recovered the *Aporodon* species groups for the ML optimality criteria, and most clades collapsed into polytomies (Appendices S4 and S5). In the *Fgb‐I7* tree, individuals from *R. mexicanus* clade IIIB were grouped within the *R. mexicanus* clade I, with generally low support values (pP ≤ 0.50, UFB ≤ 50). Specimens of the *R. mexicanus* clade II did not form a monophyletic group and their relationships with the species with which they were grouped were mostly poorly supported (pP ≤ 0.80, UFB ≤ 85). The *IRBP* tree showed minor inconsistencies with the *cytb* tree, and relationships between *Aporodon* species were, in general, better supported, compared with the *Fgb‐I7* tree, at least for the BI analysis (Appendix S5).

**FIGURE 4:**
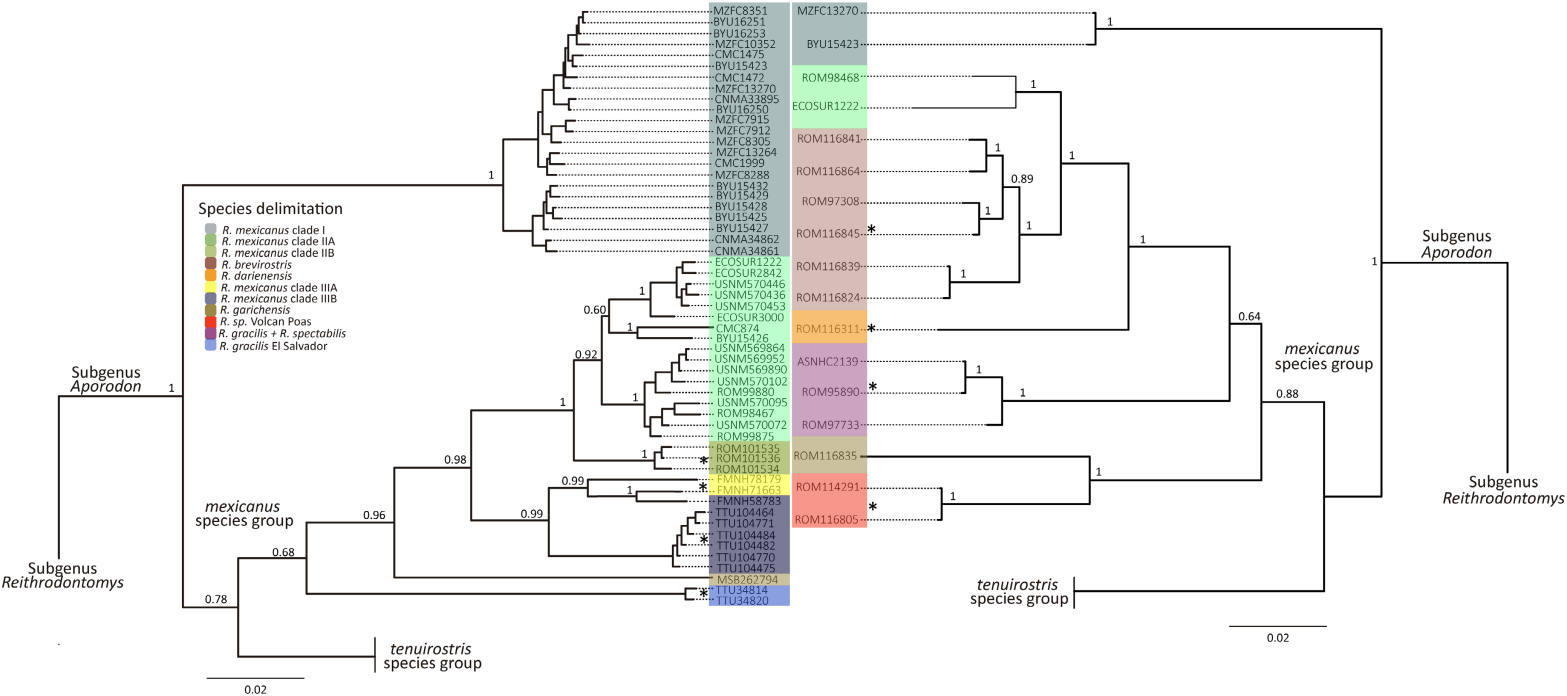
Phylogenetic relationships among delimitated species of the *Reithrodontomys mexicanus* group using sequence data of the concatenated dataset Cytochrome b + Intron 7 of the beta fibrinogen (left) and Cytochrome b + Interphotoreceptor retinoid‐binding protein (right). Values on branches represent nodal support for BI analysis. Asterisks = delimitated species not represented in both phylogenies. Terminal labels correspond to mammal collection voucher numbers (see Appendix S1).

Tree topologies obtained with the *cytb* + *Fgb‐I7* concatenated data were almost identical to the *cytb* topology (Figure 4). The only difference was in the ML analysis, where specimens of *R. gracilis* from El Salvador were grouped as the sister clade of *R. mexicanus* clade I, but with low support values (UFB = 43, Appendix S6). Phylogenies obtained with the *cytb* + *IRBP* concatenated data were generally congruent with the *cytb* tree (Figure 4). Minor differences were found with respect to the tree resulting from the ML analysis, where *R. garichensis* + *R*. sp. Volcan Poas were recovered as a sister clade to *Reithrodontomys microdon*, although these relationships were not well supported (UFB = 44, Appendix S7).

### 3.4 Geometric morphometrics of the *Reithrodontomys mexicanus* species complex

None of the skull views showed sexual dimorphism in shape (dorsal: *F* = 1.18, *p* = .28; ventral: *F* = 1.09, *p* = .36) or CS (dorsal: *t* = 0.25, *p* = .80; ventral: *t* = 0.93, *p* = .35). For the dorsal view of the skull, there was no significant correlation between CS and shape (*F* = 0.70; *p* = .55). For the ventral view, the CS‐shape correlation was significant (*F* = 8.39; *p* = .001), with CS explaining only 9.10% of the shape variation hence the allometric effect was considered relatively weak.

**FIGURE 5:**
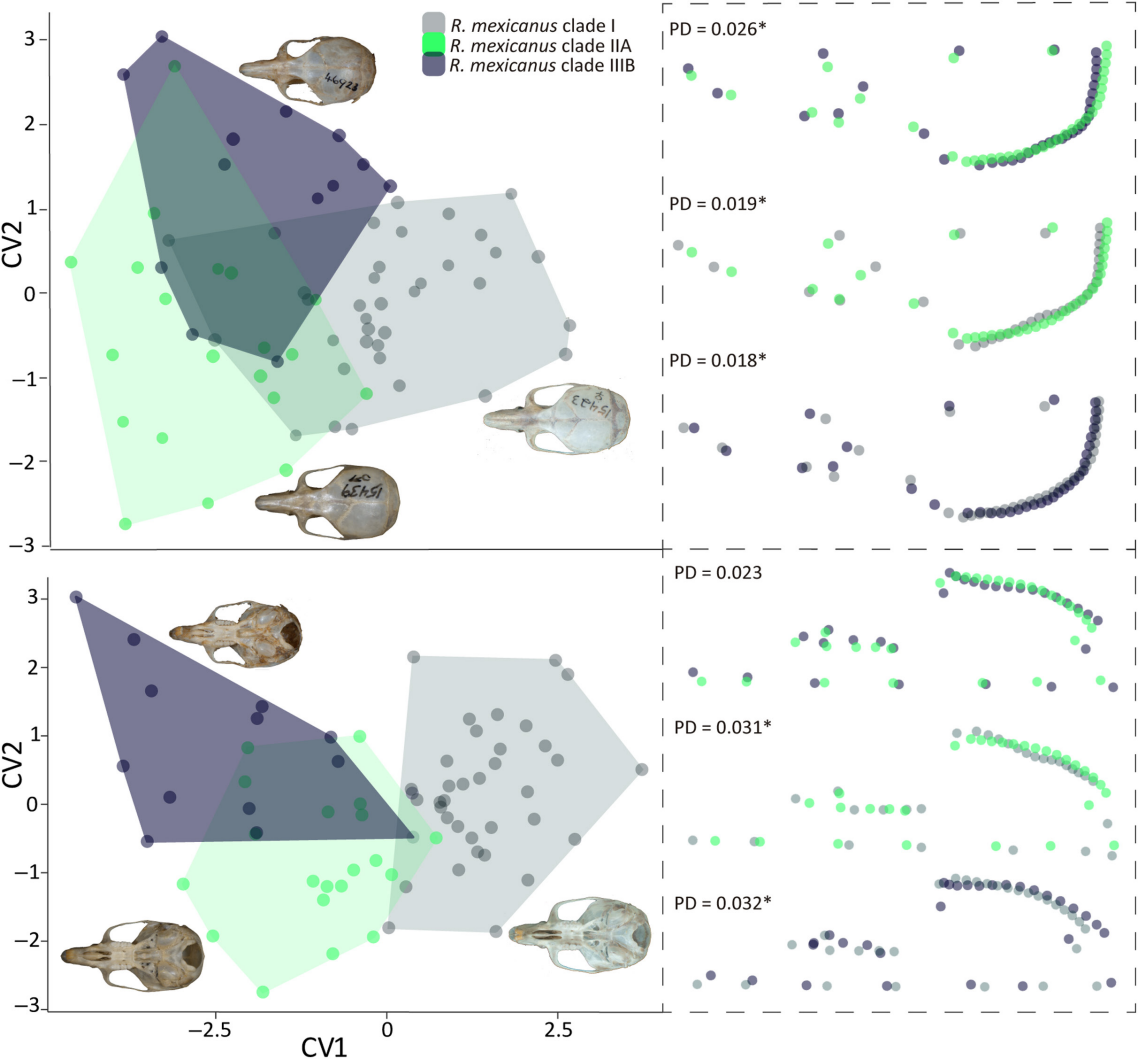
Canonical variable analysis of dorsal (top) and ventral (bottom) views of the skull for delimited species within the *Reithrodontomys mexicanus* complex. Right panel represents differences in mean shapes of each skull view, using the Procrustes distances (PD). Asterisks = significant differences based on pairwise permutation test (*p* < .05).

Significant differences were found between the cranial shape (dorsal: *F* = 3.41, *p* = .004; ventral: *F* = 8.92, *p* = .001) of the delimited species. For the dorsal view, pairwise comparisons showed significant differences among all putative species (Figure 5). The mean shape of *R. mexicanus* clade IIIB differed from *R. mexicanus* clade IIA in having a relatively longer nasal bone (landmarks 1 and 2), a slightly narrower interorbital region (landmark 5), and the braincase with a tendency to narrow toward the caudal region of the occipital bone. Compared with *R. mexicanus* clade I, the *R. mexicanus* clade IIIB showed a narrowing of the anterior‐medial region of the skull (landmarks 1–8; 35) although the braincase is slightly wider toward the ends of the parietal‐interparietal bones. *Reithrodontomys mexicanus* clade IIA and *R. mexicanus* clade I differed in most of the landmarks that characterized the skull shape, having the latter a longer rostrum and a braincase that was wider toward the parietal‐interparietal region but narrower toward the curvature of the occipital bone.

In the ventral shape, only the comparison between *R. mexicanus* clade IIA and *R. mexicanus* clade IIIB did not differ significantly in skull shape. Differences in the mean shape between *R. mexicanus* clade IIIB and *R. mexicanus* clade I occurred in the anterior region of the skull; *R. mexicanus* clade I had a longer incisive foramen, but a shorter palate. The *R. mexicanus* clade I skull shape showed a notable contraction of the landmarks 22–24, which describe the foramen magnum, with respect to the other putative species. Also, *R. mexicanus* clade I differed from *R. mexicanus* clade IIA in that the former had a shorter line of molars and slightly wider basicranium toward the posterior region of the zygomatic bar, but with a tendency to narrow toward the tympanic bullae. None of the views showed significant skull size differences (CS) between delimited species (dorsal: *F* = 2.19; *p* = .12; ventral: *F* = 1.72; *p* = .18).

The CVA showed a partial overlap in the morphospace of all putative species for the dorsal view and considerable discrimination of *R. mexicanus* clade I for the ventral view along the positive CV1 axis (Figure 5). The LDA based on skull shape did not assign 100% of the individuals to the corresponding delimited species (Table 3). The dorsal side had a lower correct discrimination rate (65%) than the ventral side (77%). In both views, most of the misclassed individuals belonged to *R. mexicanus* clade IIIB, while the best classification accuracy was in *R. mexicanus* clade I, with most individuals correctly assigned.

**TABLE 3:** Discrimination of individuals with respect to the delimited species within the *Reithrodontomys mexicanus* complex based on skull shape.

	1	2	3	%/*N*
**A. Dorsal view**				
1. *R. mexicanus* clade III	5	1	30	83.33/36
2. *R. mexicanus* clade IIA	11	5	4	55/20
3. *R. mexicanus* clade IIIB	4	5	4	30.77/13
CDR	65%			
**B. Ventral view**				
1. *R. mexicanus* clade I	2	0	34	94.44/36
2. *R. mexicanus* clade IIA	12	5	3	60/20
3. *R. mexicanus* clade IIIB	4	7	2	53.84/13
CDR	77%			

Abbreviations: CDR, correct discrimination rate in percentage; N, sample size per delimited species.

### 3.5 Ecological analyses of the *Reithrodontomys mexicanus* species complex

**FIGURE 6:**
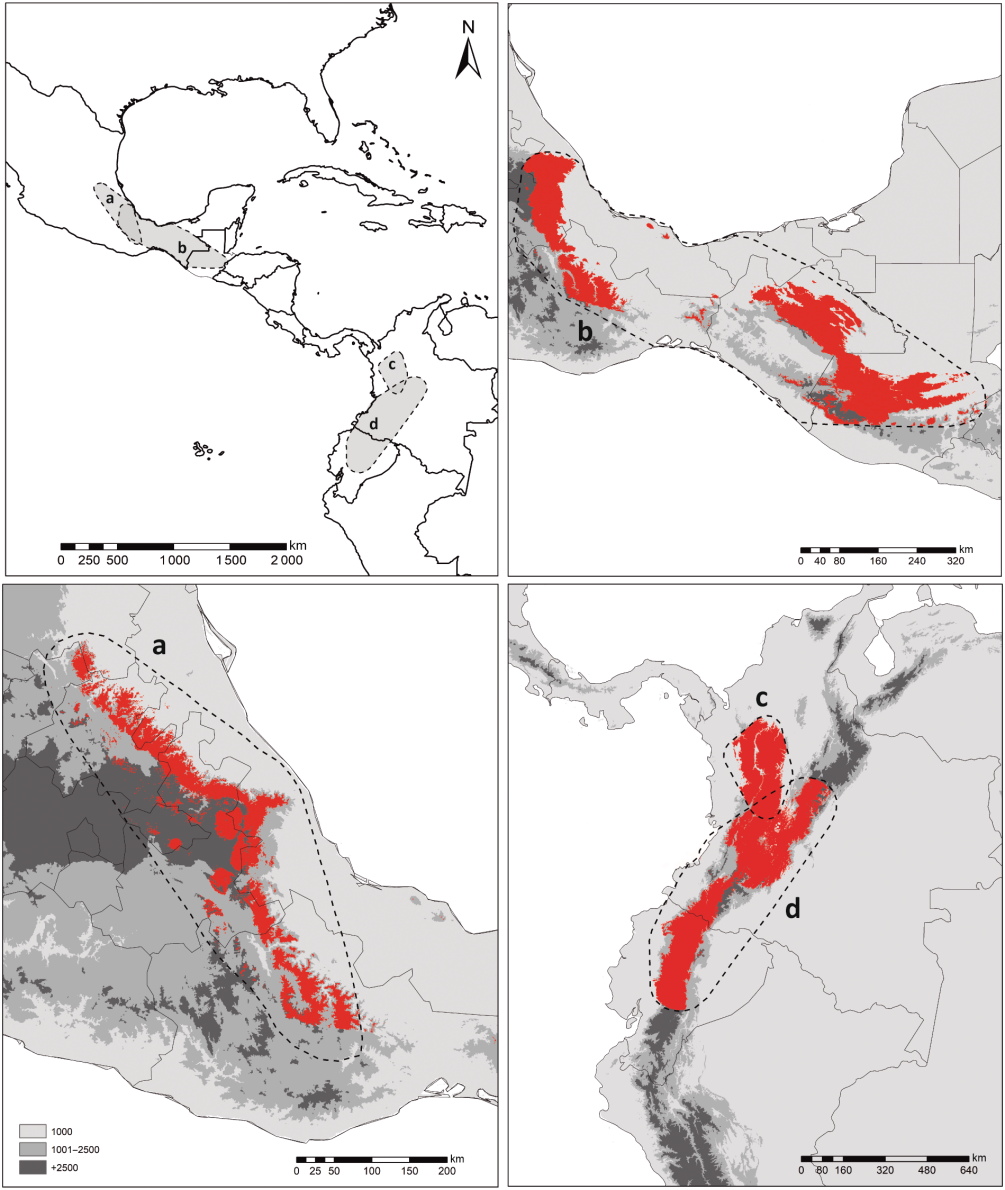
Ecological niche modeling of the delimited species within the *Reithrodontomys mexicanus* complex using six bioclimatic variables and the vegetation continuous field product. Red zones represent the high suitability areas for each species using the 10th‐percentile cutoff threshold: a = *R. mexicanus* clade I, b = *R. mexicanus* clade IIA, c = *R. mexicanus* clade IIIA, d = *R. mexicanus* clade IIIB. Calibration areas used in the construction of each final model are represented with dashed lines. Gray hues depict an elevation gradient: light gray <1000 m, gray 1000–2500 m, and dark gray >2500 m.

The partial ROC tests showed a significant predictive ability of the models for all delimited species (*p* < .05; Appendix S8). ENMs predicted high‐suitability areas for each of the delimited species (Figure 6). The *R. mexicanus* clade IIA had the largest suitability areas, mainly distributed in the Sierra Madre Oriental, northern Oaxaca, and the Central Highlands of Chiapas and Guatemala. For the *R. mexicanus* clade I, suitability areas were found mostly in the Sierra Madre Oriental and northern Oaxaca. The suitability areas of *R. mexicanus* clade IIIA were restricted to the northern region of the western and central Cordillera of the Colombian Andes, whereas the *R. mexicanus* clade IIIB occurs from the southwestern region of the Colombian Andes to the northern Ecuadorian Andes.

**FIGURE 7:**
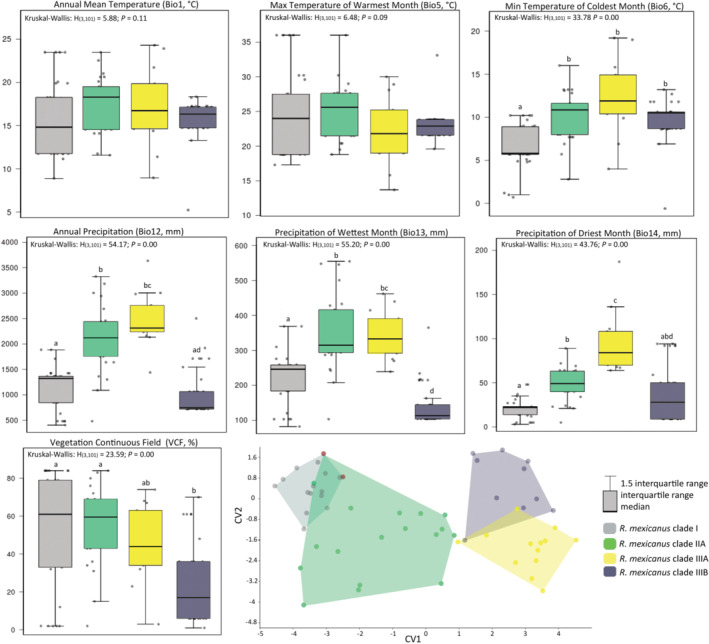
Statistical comparisons (a–g) and canonical variable analysis (h) between delimited species of the *Reithrodontomys mexicanus* complex using six bioclimatic variables and the vegetation continuous field product. Statistical significance of the post‐hoc comparisons (letters a‐d) was considered with *p* ≤ .05.

There were significant pairwise differences between the delimited species for all environmental variables except for Bio1 and Bio5 (Figure 7a‐g). The environmental niche of the *R. mexicanus* clade I presented the lowest temperature values for the coldest month (Bio6, mean of 6.5°C). The *R. mexicanus* clade IIIA presented on average the highest values of annual precipitation (Bio12, mean of 2483 mm) and precipitation of the driest month (Bio14, mean of 95.2 mm) but showed similar average values of precipitation of the wettest month, together with the *R. mexicanus* clade IIA (Bio13, mean of 340.3 y 345.9 mm, respectively). The CVA completely segregated the environmental niches of *R. mexicanus* clade IIIA–*R. mexicanus* clade IIIB from those of *R. mexicanus* clade I–*R. mexicanus* clade IIA, but there was still a small region of overlap in their environmental space (Figure 7h).

## 4 DISCUSSION

### 4.1 *Reithrodontomys mexicanus* cryptic species complex

*Reithrodontomys mexicanus* originally comprised 13 subspecies (Hooper, 1952, 1955), though 10 are currently recognized (Bradley, 2017). Our phylogenies included samples of the formerly subspecies *R. garichensis* for the first time. We confirmed that it is distinct from *R. mexicanus* but belongs to the *R. mexicanus* group (Gardner & Carleton, 2009). Surprisingly, the Costa Rican specimens considered to be *Reithrodontomys* sp. by Miller and Engstrom (2008) showed a close relationship with *R. garichensis*. These authors suggested that the two specimens from Volcan Poas, Costa Rica represent a new species based on their morphological and genetic differences with respect to *R. mexicanus*. The three species delimitation methods implemented here supported their proposal. On the other hand, these methods failed to differentiate the specimen from La Carpentera, Costa Rica from that of *R. garichensis*. Therefore, both were identified as the same species, and the specimen from La Carpentera, Costa Rica is proposed to be reclassified as *R. garichensis*.

In this study, *R. mexicanus* was recovered as a polyphyletic taxon formed by three clades (I, II, III), highly divergent from each other, which agrees with Arellano et al. (2003, 2005). Within clade II, all delimitation methods demarcated individuals from Parque Nacional Montecristo, El Salvador (clade IIB) to the species level. The *cytb* genetic distance between this group and clade IIA was 4.96%, slightly lower than the 5% limit estimated by Baker and Bradley (2006) to recognize sister mammal species. Specimens from El Salvador could not be examined morphologically. Nonetheless, we had access to five individuals from Los Esesmiles (Cerro El Pital); not included in the molecular or morphometric analyses. Compared with individuals of *R. mexicanus* clade I distributed in Mexico and Guatemala, these specimens from Los Esesmiles differ morphologically by their relatively shorter nasal bones, broader palatal, and rounded braincase. The pelage exhibits a cinnamon coloration, and the tail tends to be not much longer than the head and body together. Parque Nacional Montecristo and Los Esesmiles are only separated by ca. 30 km, so individuals from both localities could be assumed to have the same genetic identity. Thus, populations from El Salvador could be assumed to be a divergent lineage from the remaining members of *R. mexicanus* clade IIA. However, due to the limited geographic sampling and the lack of additional evidence, such as those used in this study, we suggest keeping *R. mexicanus* clade IIB as part of *R. mexicanus* sensu stricto but highlighting the need of additional studies to reach a taxonomic conclusion regarding populations from El Salvador.

The strongest morphometric similarities were found between the putative species *R. mexicanus* clade IIA and *R. mexicanus* clade IIIB, which showed no significant differences in the ventral skull shape. It has been reported that the ventral view of the skull is the one with a better phylogenetic signal (Camul & Polly, 2005). Consequently, our morphometric results are consistent with the molecular data, which showed that these clades are more closely related to each other than to the *R. mexicanus* clade I. In mammals, it has been reported that the environment can influence the development of bone structures such as the skull and jaw (Camul & Polly, 2005). The few morphometric differences found between these putative species (clades IIA and IIIB) could be because, in general, they share similar habitat characteristics (Hooper, 1952). However, the ecological analyses allowed us to clearly segregate their environmental space, based mainly on precipitation and VCF variables. Although their separation was weakly supported by geometric morphometrics, the genetic and environmental differentiation of these clades strongly support their demarcation as distinct species.

The bGMYC delimitation method proved to be the most conservative for proposing putative species within the clade that grouped the *R. mexicanus* specimens from South America. Despite the number of species proposed by the different methods, most pairwise comparisons did not exceed *cytb* genetic distance values of 5% (K2P distances range from 1.50% to 4.20%, Appendix S3). Low levels of genetic differentiation have been reported in South American rodents, which have been explained because of recent speciation processes (Patton & Smith, 1992). Such would be the case in the genus *Reithrodontomys*, whose diversification processes began ca. 6.83 mya according to our results, expanding from North America to South America, of which only subspecies of *R. mexicanus* are known (Hooper, 1952).

Arellano et al. (2005) analyzed a specimen from Colombia but retained it as part of *R. mexicanus* sensu stricto. In their analyses, *R. darienensis* from Panama was not included. Our phylogenetic analyses included *R. darienensis* and a good representation of specimens from Colombia and Ecuador that had been classified a priori as *R. mexicanus*. Our evidence supported the conclusion that South American specimens form a distinct clade from *R. mexicanus* which is sister to *R. darienensis*. Within this clade, delimitation methods and *cytb* genetic distances suggested that the Colombian specimens (*R. mexicanus* clade IIIA) from Risaralda (ICN16579) and Antioquia (FMNH78179) constitute a distinct species from the other South American individuals. Poorly supported phylogenetic relationships (by ML and BI methods) between these putative species could be additional evidence of recent speciation processes. However, different sources of genetic information and extensive geographic sampling are needed to obtain a more comprehensive understanding of the evolutionary history of these South American rodents.

Although it was not possible to corroborate the species‐level distinction of the *R. mexicanus* clade IIIA with morphological evidence, the ENM delimited its distribution to the northwestern region of the western and central Cordilleras of Colombia, with a habitat characterized mainly by high precipitation values. These individuals could be considered a divergent lineage that is already distinct from the rest of the South American populations in at least three species properties (reciprocal monophyly, genetic differentiation, and ecological niche distinctiveness; de Queiroz, 2007).

The remaining individuals from Colombia and Ecuador (*R. mexicanus* clade IIIB) were validated at the species level by genetic and ecological data, and to a lesser extent with morphometric evidence. Hooper (1952) reported three subspecies of *R. mexicanus* in South America: *R. m. milleri*, *R. m. soederstroemi*, and *R. m. eremiscus*. The known distribution of *R. m. milleri* ranges from Colombia to northern Ecuador, including the suitability areas found for the two candidate species (*R. mexicanus* clade IIIA and *R. mexicanus* clade IIIB). However, the distribution of *R. mexicanus* clade IIIA was restricted to a small region of the western and central Cordillera, whereas *R. mexicanus* clade IIIB was distributed mainly in the Cordillera Oriental, a region not reported for *R. m. milleri* (Hooper, 1952). In addition, the suitability areas of *R. mexicanus* clade IIIB included localities recognized for the other two subspecies (*R. m. soederstroemi* and *R. m. eremiscus*), distributed only in Ecuador (Arellano, 2015; Hooper, 1952). Therefore, an analysis focused on the harvest mice populations of South America is essential to correctly establish the taxonomic designation of the *Reithrodontomys* species that inhabit this region because they undoubtedly do not belong to *R. mexicanus*.

Even though *R. mexicanus* clade I had already been considered a candidate species using allozymes (Arellano et al., 2003), *cytb* sequences (Arellano et al., 2005), and chromosomal data (Urbina et al., 2006), in our analyses, we were able to include a wide sampling that allowed us to support this new species not only with molecular data but also with morphological and ecological data. The phylogenetic position of this putative species in the trees confirms that it is a much older lineage (divergence time estimates ranging from 4.53 to 6.84 mya) and has a different evolutionary history from the rest of the clades within the subgenus *Aporodon*. Although representatives of the *R. mexicanus* clade I have historically been classified as *R. mexicanus*, they are genetically very distant from this species, even those that coexist in sympatry in the localities of La Esperanza and Puerto de la Soledad in Oaxaca, Mexico. Specimens of this clade could be discriminated correctly by morphology, especially by ventral skull shape. The phylogenetic signal that structures located on the ventral side of the skull exhibit (Lockwood et al., 2004; Macholán, 2008) could explain the marked morphometric differentiation that this clade presented in accordance with its position in the phylogenetic trees. The environmental characteristics of this candidate species, partially overlap with those of *R. mexicanus* clade II. This is expected, given that they share part of their distribution in the Mexican cloud forests (Gual–Díaz & Rendón‐Correa, 2014). However, comparisons of most environmental variables were significantly different, with *R. mexicanus* clade I occupying a geographic area characterized by low values of temperatures and annual precipitation, and high forest cover (VCF). The congruence between the independent datasets is essential for the delimitation of this clade as a new species since until now, only molecular data had been used to differentiate it.

### 4.2 Taxonomy of the *Reithrodontomys mexicanus* species group

Within the subgenus *Aporodon*, the *R. mexicanus* group currently comprises the species *R. mexicanus*, *R. brevirostris*, *R. paradoxus*, *R. gracilis, R. spectabilis*, *R. darienensis*, and *R. garichensis*. The overall distribution of this group ranges from Mexico to South America, although most species are concentrated in Central America (Hall, 1981). Here, all the members of this group, but *R. paradoxus*, were analyzed using molecular data, which allowed us to clarify the evolutionary relationships between them and make taxonomic considerations within the species group.

*Reithrodontomys brevirostris* was recovered as the sister group of the *R. mexicanus* clade II and confirmed as a valid species by all molecular delimitation methods. However, populations of this species have tended to be confused with those of *R. mexicanus* from Central America. Indeed, most of the individuals that exemplified the *R. brevirostris* clade in this study had originally been identified by their collectors as *R. mexicanus* (and another two as *R. gracilis* by Miller & Engstrom, 2008). Similarly, in the phylogeny of Arellano et al. (2005) one individual from Costa Rica, grouped in their Clade I, was later reclassified as *R. brevirostris* by Gardner and Carleton (2009). Furthermore, these later authors assigned *R. m. potrerograndei*, a former *R. mexicanus* subspecies, as part of *R. brevirostris* “because of their comparably small size and other morphological resemblances” (Gardner & Carleton, 2009: 172). Hooper (1952) noted that many of the morphological and cranial features of *R. brevirostris* were reminiscent of *R. mexicanus*, but the absence of evidence of interbreeding allowed them to be maintained as species. The separation between *R. brevirostris* and *R. mexicanus* clade II occurred at approximately 1.49 mya, and its genetic divergence for *cytb* was 5.68%, slightly higher than 5%, a generally observed distance between sister species in mammals (Baker & Bradley, 2006). The relatively low genetic differentiation could account for the strong morphological similarity historically reported between these clades. They also share similar habitat characteristics, being distributed mainly in the cloud forest (Gual–Díaz & Rendón‐Correa, 2014; Hooper, 1952). Both clades would fall within the gray zone described by de Queiroz (2007), in which the decision as to whether they constitute one or two taxonomic entities depends on the species criteria used. Based on our results, we propose that they remain distinct entities, under the assumption that they have been evolving as divergent lineages for sufficient time to separate but continue to maintain many of the common ancestral characteristics they share (de Queiroz, 1998).

Hooper (1952) considered *R. darienensis* and *R. gracilis* to be superspecies because they did not show major differences in morphological traits, pelage coloration, or cranial or body size. The term superspecies was proposed by B. Rensch and later by E. Mayr to refer to monophyletic and allopatric taxa that formed a single entity and later evolved to the species level (Amadon, 1966). However, our phylogenetic results did not recover *R. darienensis* and *R. gracilis* as a monophyletic group, and the genetic distances between them reached values of almost 14%. Furthermore, *R. darienensis* was more closely related to the clade containing *R. mexicanus* specimens from South America (although they were genetically well differentiated) than to *R. gracilis*. This is consistent with its restricted distribution in eastern Panama (Bradley, 2017). The *R. gracilis* specimens from Yucatan and Campeche, Mexico were unequivocally delimited as the same entity as *R. spectabilis*, while those from El Salvador were recognized at the species level. According to their geographical distribution, these two specimens from El Salvador correspond to the subspecies *R. g. anthonyi* (Hall, 1981), but the genetic distances of almost 8% between these and the *R. gracilis* + *R. spectabilis* clade suggest that it is necessary to reevaluate the Central American populations of *R. gracilis*, to assess if they completed speciation processes (Futuyma, 2013).

*Reithrodontomys spectabilis*, whose distribution is restricted to Cozumel Island, Mexico, was described as one of the largest species of the genus (Jones Jr. & Lawlor, 1965). Although many aspects of its morphology were reminiscent of *R. gracilis* from Yucatan, marked differences in body size, darker coloration, and broader and heavier zygomatic arches prompted its recognition at the species level. Jones Jr. and Lawlor (1965) suggested that the precursor of *R. spectabilis* arrived from the Yucatan Peninsula during the Late Pleistocene, which assumes a relatively long period of isolation between these two species. Our results suggest that the divergence between these species (95% HPD = 0.19–0.47) occurred at some point in the Middle Pleistocene (from 0.781 to 0.126 Mya, Walker et al., 2018), indicating a very recent separation between their populations compared with those reported for other species of the subgenus *Aporodon* (Martínez‐Borrego, Arellano, Cruz, et al., 2022; this study). This recent separation is also consistent with the low *cytb* genetic differentiation between these species (0.7%), which fall within the intraspecific range values proposed for *Reithrodontomys* (Baker & Bradley, 2006). The phylogenetic relationships between *R. spectabilis* and *R. gracilis* have been analyzed in the past with allozymes and the *cytb* gene, arriving at similar results to ours and suggesting an island effect as a possible cause of their morphological differences (Arellano et al., 2003, 2005). Rodents frequently exhibit island gigantism with respect to conspecific populations on the mainland (Lomolino, 1985). This phenomenon is known as the Island rule (Foster, 1964) and is affected by different factors including resources availability and the absence of natural predators (Lomolino, 2005). We agree with Arellano et al. (2005) that this phenomenon could explain why the harvest mice populations of Isla Cozumel differ, mainly in body size, from the *R. gracilis* population*s* of Yucatan. Recognizing *R. spectabilis* as a conspecific of *R. gracilis* entails reevaluating its populations in many ways, considering that it is an endemic species classified as Critically Endangered by the IUCN Red List of Threatened Species (Vázquez et al., 2018). Additional comparative studies of both species employing different sources of evidence, such as geometric morphometric, ecological niche, and population genetics, among others, are necessary to reach a conclusion about their taxonomic status.

### 4.3 Species delimitation and its taxonomic implications

Establishing species boundaries is difficult when dealing with taxonomically complex groups whose descriptions have been based primarily on their morphology (Dayrat, 2005). Many of these species exhibit such a pronounced morphological resemblance to each other that they are recognized as cryptic species (Bickford et al., 2007). This is the case of *R. mexicanus*, where molecular (Arellano et al., 2003, 2005; Miller & Engstrom, 2008) and craniodental (Gardner & Carleton, 2009) data have revealed broad cryptic variation leading to its recognition as a species complex. Integrating multiple approaches to delimit species has been strongly recommended to better confirm the species hypothesis (Dayrat, 2005; Will et al., 2005). In this study, we implemented an integrative taxonomy approach to test if there are cryptic lineages within *R. mexicanus* that are evolving separately. We employed different criteria in accordance with the GLC (de Queiroz, 1998, 2007), seeking as much evidence as possible to support the recognition of identified candidate species (Sangster, 2018).

Species proposals were not always congruent among the delimitation methods. The limitations of methods based on DNA data have been discussed previously (see Luo et al., 2018), mainly those related to noncompliance with the assumptions of the method (Carstens et al., 2013). However, in *Reithrodontomys*, the efficacy of the delimitation methods used here (mPTP, bGMYC, and STACEY) to demarcate cryptic lineages at the species level has been demonstrated (Martínez‐Borrego, Arellano, Cruz, et al., 2022). The use of different molecular markers showed conflicting results among the phylogenies, with a high discordance between the topology of the *cytb* and those of the *Fgb‐I7* and *IRBP*, respectively. This mitonuclear discordance has been suggested to be a consequence of introgression or incomplete lineage sorting (Firneno Jr. et al., 2020; Toews & Brelsford, 2012). Although including more genetic markers can help to elucidate species limits, sometimes the use of multiple loci has complicated this purpose for taxonomists (Firneno Jr. et al., 2021). Therefore, in our study, the recognition of taxonomic entities was based primarily on molecular species delimitation methods, including the *cytb* genetic distances, traditionally used in mammal genetic studies (Baker & Bradley, 2006; Bradley & Baker, 2001), but other evidence such as skull morphometry and ecological niche was also used.

Geometric morphometrics and niche modeling have shown great applicability in taxonomic studies in mammals (Barčiová, 2009; Martínez‐Gordillo et al., 2010) and have allowed the corroboration of species limits hypotheses derived from phylogenetic studies (e.g., Camul & Polly, 2005; Rivera et al., 2018). However, the species‐level clades proposed here by the three delimitation methods and genetic distances were not always strongly supported by ecological and/or morphological data. Under the GLC, failure to meet any of the species criteria does not necessarily mean that the candidate species does not correspond to a divergent lineage (de Queiroz, 2007). Rather, GLC recognizes that species properties may evolve at different times during divergence (Sangster, 2018), hence the importance of integrating multiple data sources to support or reject the species hypothesis (Padial et al., 2010).

## 5 CONCLUSIONS

This work confirms that *R. mexicanus* sensu lato is a cryptic species complex composed of at least four putative species: *R. mexicanus* clade I, *R. mexicanus* clade IIA (*R. mexicanus* sensu stricto), *R. mexicanus* clade IIIA, and *R. mexicanus* clade IIIB. In addition, specimens from El Salvador (*R. mexicanus* clade IIB) should be reevaluated taxonomically including a better sampling of multiple lines of evidence. For *R. mexicanus* sensu stricto, additional analyses are necessary to estimate its phylogenetic relationship with respect to the subspecies *R. mexicanus riparius*, which was not included in our analyses but has evident geographic isolation from the other Mexican populations (Hall, 1981; Hooper, 1955). Finally, *R. mexicanus* clade I constitute a new species, pending formal description and assignment of a scientific name according to the International Code of Zoological Nomenclature rules.

## AUTHOR CONTRIBUTIONS

**Daily Martínez‐Borrego:** Conceptualization (equal); data curation (lead); formal analysis (lead); investigation (lead); methodology (lead); writing – original draft (lead). **Elizabeth Arellano:** Conceptualization (equal); methodology (supporting); resources (lead); supervision (lead); writing – review and editing (supporting). **Francisco X. González‐Cózatl:** Conceptualization (equal); methodology (supporting); supervision (supporting); writing – review and editing (supporting). **Sandra M. Ospina‐Garcés:** Conceptualization (equal); methodology (supporting); writing – review and editing (supporting). **Duke S. Rogers:** Conceptualization (equal); methodology (supporting); resources (supporting); writing – review and editing (supporting).

## FUNDING INFORMATION

DM‐B was supported by a Ph.D. Scholarship Program 2018‐000012 01NACF‐11852LANC from Consejo Nacional de Ciencia y Tecnología (CONACYT), Mexico. Fieldwork season 2019–2020 was carried out thanks to the Latin American Field Research Award 2019 from the American Society of Mammologists.

## CONFLICT OF INTEREST STATEMENT

The authors declare that they have no competing interests.

## Supporting information


Appendix S1.

Appendix S2.

Appendix S3.

Appendix S4.

Appendix S5.

Appendix S6.

Appendix S7.

Appendix S8.

## Data Availability

DNA sequences: GenBank accession numbers ON156861–ON156912 (*cytb*), ON156913–ON156967 (*Fgb‐I7*), and ON156968–ON156971 (*IRBP*). Parameter selection for the construction of ecological niche models: FigShare doi: https://10.6084/m9.figshare.22190878. Occurrence points and ecological data: FigShare doi: https://10.6084/m9.figshare.22194610.
