# Supplementary material for: Species delimitation and integrative taxonomy of the Reithrodontomys mexicanus (Rodentia: Cricetidae) cryptic complex
Source: Ecol Evol. 2023 Jul 30;13(8):e10355. doi: 10.1002/ece3.10355 (PMC10387591; doi:10.1002/ece3.10355)
Supplement: Supplementary file 1 — Appendix S1. [file ECE3-13-e10355-s003.pdf]

Appendix 1

List of *Reithrodontomys* specimens used in the molecular analysis and geometric morphometric and ecological analyses (in the case of *R. mexicanus*). Collecting localities and GenBank accessions numbers for Cytochrome *b*, Intron 7 of the beta fibrinogen, and Interphotoreceptor retinoid-binding protein genes are given (GenBank accessions highlighted in bold represent DNA sequences generated in this study). Abbreviations for analysis, countries, and mammal collections housing the specimens we included are as follows: E = ecology; GM = geometric morphometric; M = molecular; COL = Colombia; CR = Costa Rica; ECU = Ecuador; ES = El Salvador; GU = Guatemala; MX = Mexico; NIC = Nicaragua; PAN = Panama; AMNH = American Museum of Natural History; ASNHC = Angelo State Natural History Collections; BYU = Brigham Young University; CMC = Colección de Mamíferos del CIByC; CNMA = Colección Nacional de Mamíferos, Universidad Nacional Autónoma de México; ECOSUR = Colección de Mamíferos de El Colegio de la Frontera Sur, San Cristóbal; FMNH = Field Museum of Natural History; ICN = Colección de Mamíferos, Instituto de Ciencias Naturales; LSUMNS = Louisiana State University Museum of Zoology; MVZ = Museum of Vertebrate Zoology; MZFC = Colección de Mamíferos, Museo de Zoología "Alfonso L. Herrera", Universidad Nacional Autónoma de México; ROM = Royal Ontario Museum; TTU = Mammal Collection, Texas Tech University; UMMZ = Museum of Zoology, University of Michigan; USNM = Division of Mammals, National Museum of Natural History; UV = Colección de Mamíferos, Universidad Veracruzana.

Current species name / Voucher	Locality	Analysis	GenBank Accession		
			cytb	Fgb-I7	IRBP
R. mexicanus AMNH 32582	COL: Popayán, Cerro Munchique, Cauca.	GM			
R. mexicanus AMNH 32584	COL: Popayán, Cerro Munchique, Cauca.	GM			
R. mexicanus AMNH 32590	COL: Popayán, Cerro Munchique, Cauca.	GM			
R. mexicanus AMNH 32599	COL: El Tambo, Cocal, Cauca.	GM			
R. mexicanus AMNH 46908	ECU: Quito, Pichincha.	GM			
R. mexicanus AMNH 46920	ECU: Quito, Pichincha.	GM			
R. mexicanus AMNH 46923	ECU: Quito, Pichincha.	GM			
R. mexicanus AMNH 46977	ECU: Santo Domingo, Alobuela, Pichincha.	GM			
R. mexicanus AMNH 46978	ECU: Santo Domingo, Alobuela, Pichincha.	GM			
R. mexicanus AMNH 46980	ECU: Santo Domingo, Alobuela, Pichincha.	GM			

R. mexicanus AMNH 46982	ECU: Santo Domingo, Alobuela, Pichincha.	GM			
R. mexicanus AMNH 46997	ECU: Quito, Perucho, Pichincha.	GM			
R. mexicanus AMNH 47003	ECU: Guayllabamba River, Pichincha.	GM			
R. mexicanus BYU15432	MX: 28 km SW (by road.) La Esperanza, Municipio Santiago Comaltepec, Oaxaca.	M, E	AY859435	**ON156946**	
R. mexicanus BYU15429	MX: 11 km SW (by road.) La Esperanza, Municipio Santiago Comaltepec, Oaxaca.	M, GM, E	AY859444	**ON156933**	
R. mexicanus BYU15431	MX: 11 km SW (by road.) La Esperanza, Municipio Santiago Comaltepec, Oaxaca.	M, GM, E	AY859446		
R. mexicanus BYU15427	MX: 11 km SW (by road.) La Esperanza, Municipio Santiago Comaltepec, Oaxaca.	M, GM, E	AY859442	**ON156936**	
R. mexicanus BYU15430	MX: 11 km SW (by road.) La Esperanza, Municipio Santiago Comaltepec, Oaxaca.	M, GM, E	AY859445		
R. mexicanus BYU15424	MX: 11 km SW (by road.) La Esperanza, Municipio Santiago Comaltepec, Oaxaca.	M, GM, E	AY859434		
R. mexicanus BYU15428	MX: 11 km SW (by road.) La Esperanza, Municipio Santiago Comaltepec, Oaxaca.	M, GM, E	AY859443	**ON156927**	
R. mexicanus BYU15425	MX: 11 km SW (by road.) La Esperanza, Municipio Santiago Comaltepec, Oaxaca.	M, GM, E	AY859441	**ON156947**	
R. mexicanus BYU16250	MX: 1.5 km S Puerto de la Soledad, Municipio Teotitlán de Flores Magón, Oaxaca.	M, GM, E	AY859437	**ON156945**	
R. mexicanus BYU16251	MX: 1.5 km S Puerto de la Soledad, Municipio Teotitlán de Flores Magón, Oaxaca.	M, GM, E	AY859438	**ON156941**	
R. mexicanus BYU16252	MX: 1.5 km S Puerto de la Soledad, Municipio Teotitlán de Flores Magón, Oaxaca.	M, GM, E	AY859439		
R. mexicanus BYU16253	MX: 1.5 km S Puerto de la Soledad, Municipio Teotitlán de Flores Magón, Oaxaca.	M, GM, E	AY859440	**ON156942**	
R. mexicanus BYU15423	MX: 6 km S Zacualtipán, Rancho la Mojonera, Municipio Zacualtipán, Hidalgo.	M, GM, E	AY859433	**ON156948**	**ON156971**
R. mexicanus BYU15439	MX: 18 km NW Teocelo, Municipio Ixhuatlán, Veracruz.	M, GM, E	AY293822		
R. mexicanus BYU15436	MX: 1.5 km S Puerto de la Soledad, Municipio Teotitlán de Flores Magón, Oaxaca.	M, GM, E	AY859448		

R. mexicanus BYU15426	MX: 11 km SW (by road.) La Esperanza, Municipio Santiago Comaltepec, Oaxaca.	M, GM, E	AY859449	MW117093	
R. mexicanus BYU20781	MX: Rancho La Providencia, Chiapas.	M, GM, E	HQ269733		
R. mexicanus CMC852	MX: Xometla (ravine over the bridge), Municipio La Perla, Veracruz.	M, GM, E	**ON156862**		
R. mexicanus CMC1472	MX: 3.4 km SW from desviation to Mazatepec, Mesa de la Yerba, Veracruz.	M, GM, E	**ON156863**	**ON156937**	
R. mexicanus CMC1475	MX: Matlalapa, Municipio Xico, Veracruz.	M, GM, E	**ON156865**	**ON156939**	
R. mexicanus CMC841	MX: 2.9 km E Puerto del Aire, Municipio Acultzingo, Veracruz.	M, GM, E	**ON156868**		
R. mexicanus CMC1999	MX: Rancho 22 de marzo, km 75.8 Ahuazotepec-Zacatlán road, Puebla.	M, GM, E	**ON156872**	**ON156934**	
R. mexicanus CMC872	MX: 1.9 km N Las Cañadas (Eco reserve), Municipio Huatusco, Veracruz.	M, GM, E	**ON156879**		
R. mexicanus CMC874	MX: 1.9 km N Las Cañadas (Eco reserve), Municipio Huatusco, Veracruz.	M, GM, E	HQ269734	HQ269796	
R. mexicanus CMC877	MX: 1.9 km N Las Cañadas (Eco reserve), Municipio Huatusco, Veracruz.	GM, E			
R. mexicanus CMC842	MX: 2.9 km E Puerto del Aire, Municipio Acultzingo, Veracruz.	GM, E			
R. mexicanus CMC844	MX: 2.9 km E Puerto del Aire, Municipio Acultzingo, Veracruz.	GM, E			
R. mexicanus CMC845	MX: 2.9 km E Puerto del Aire, Municipio Acultzingo, Veracruz.	GM, E			
R. mexicanus CMC846	MX: 2.9 km E Puerto del Aire, Municipio Acultzingo, Veracruz.	GM, E			
R. mexicanus CMC865	MX: Xometla (ravine over the bridge), Municipio La Perla, Veracruz.	GM, E			
R. mexicanus CMC868	MX: Xometla (ravine over the bridge), Municipio La Perla, Veracruz.	GM, E			
R. mexicanus CMC1377	MX: 3.4 km SW from desviation to Mazatepec, Mesa de la Yerba, Veracruz.	GM, E			
R. mexicanus CNMA34862	MX: 5 km N Cerro Zempoaltepelt, Santa María Yacochi, Oaxaca.	M, GM, E	**ON156860**	**ON156929**	
R. mexicanus CNMA33895	MX: Puerto de la Soledad, Municipio Teotitlán de Flores Magón, Oaxaca.	M, GM, E	AY859436	**ON156932**	

R. mexicanus CNMA42279	MX: Rancho La Providencia, Unión Juárez, Chiapas.	M, E	AY859450		
R. mexicanus CNMA34861	MX: 5 km N Cerro Zempoaltepelt, Santa María Yacochi, Oaxaca.	M, GM, E	**ON156874**	**ON156928**	
R. mexicanus ECOSUR1222	MX: 3.45 km N El Vivero, Las Grutas, Lagos de Montebello National Park, Chiapas.	M, E	**ON156880**	**ON156964**	**ON156968**
R. mexicanus ECOSUR2842	MX: Mercado Indígena, Santo Tomás Oxchuc, Chiapas.	M, GM. E	**ON156881**	**ON156952**	
R. mexicanus ECOSUR3000	MX: Ejido Sombra Chica, 1 km NW Tumbalá, Chiapas.	M, GM, E	**ON156883**	**ON156962**	
R. mexicanus ECOSUR931	MX: 3.45 km N El Vivero, Las Grutas, Lagos de Montebello National Park, Chiapas.	GM			
R. mexicanus FMNH78179	COL: Las Palmas, Medellín, Antioquia.	M, E	**ON156896**	**ON156925**	
R. mexicanus FMNH90343	COL: 650m Guachicono River, Cauca.	M, E	**ON156897**		
R. mexicanus FMNH71663	COL: San Cristóbal, Cundinamarca, Bogotá.	M, E	**ON156901**	**ON156935**	
R. mexicanus FMNH58783	COL: Las Cuevas Parque, Upper Cabana, cocina, Huila.	M, E	**ON156902**	**ON156931**	
R. mexicanus FMNH89348	COL: Charguayaco, Cauca.	M, E	**ON156903**		
R. mexicanus FMNH71680	COL: Guapantal, Urrao, Antioquia.	E			
R. mexicanus FMNH71666	COL: Paramo Frontino, Urrao, Antioquia.	E			
R. mexicanus FMNH71666	COL: 15 km E Negrito River, Sonson, Antioquia.	E			
R. mexicanus FMNH71658	COL: Termales River, Manizales, Caldas.	E			
R. mexicanus FMNH71685	COL: Santa Barbara, Urrao, Antioquia.	E			
R. mexicanus FMNH71686	COL: Urrao River, Urrao, Antioquia.	E			
R. mexicanus FMNH70166	COL: Valdivia, Quebrada Valdivia, Antioquia.	E			
R. mexicanus FMNH69653	COL: Santa Elena, Medellín, Antioquia.	E			
R. mexicanus ICN16579	COL: Vereda La Pastora, road to Las Cascadas, PRN Ucumar, Corregimiento La Florida, Municipio Pereira, Risaralda.	M, E	AF108708		
R. mexicanus ICN16097	COL: 4 km S, Vereda Puente Peláez, farm Cañaveral, Retiro, Antioquia.	E			
R. mexicanus ICN16760	COL: Vereda El Tambo, farm Cde. Margarita Molina., La Unión, Antioquia.	E			

R. mexicanus ICN11684	COL: Vereda Cocora, Estación Forestal, farm La Montaña, Salento, Quindío.	E			
R. mexicanus MVZ139529	ECU: 22 km NNE Quito, Pichincha.	E			
R. mexicanus MVZ 139530	ECU: 26 km NNE Quito, Pichincha.	E			
R. mexicanus MZFC7915	MX: El Pemoche, Landa de Matamoros, Querétaro.	M, GM, E	**ON156861**	**ON156940**	
R. mexicanus MZFC10352	MX: Puerto de la Soledad, Municipio Teotitlán de Flores Magón, Oaxaca.	M, GM, E	**ON156864**	**ON156938**	
R. mexicanus MZFC8351	MX: Puerto de la Soledad, Municipio Teotitlán de Flores Magón, Oaxaca.	M, GM, E	**ON156867**	**ON156926**	
R. mexicanus MZFC13270	MX: Xochititan, Puebla.	M, GM, E	**ON156869**	**ON156949**	**ON156970**
R. mexicanus MZFC8305	MX: 6 km NE Zacualpan, Municipio Zacualpan, Estado de México.	M, GM, E	**ON156870**	**ON156944**	
R. mexicanus MZFC13264	MX: Xochititan, Puebla.	M, GM, E	**ON156871**	**ON156930**	
R. mexicanus MZFC8288	MX: 6 km NE Zacualpan, Municipio Zacualpan, Estado de México.	M, GM, E	**ON156873**	**ON156950**	
R. mexicanus MZFC7912	MX: El Pemoche, Landa de Matamoros, Querétaro.	M, GM, E	**ON156875**	**ON156943**	
R. mexicanus ROM101534	ES: Montecristo National Park, Los Planes, Santa Ana.	M	**ON156876**	**ON156961**	
R. mexicanus ROM101535	ES: Montecristo National Park, Los Planes, Santa Ana.	M	**ON156877**	**ON156963**	
R. mexicanus ROM101536	ES: Montecristo National Park, Los Planes, Santa Ana.	M, E	**ON156878**	**ON156960**	
R. mexicanus ROM101508	ES: Montecristo National Park, Los Planes, Santa Ana.	M, E	AY859453		
R. mexicanus ROM97543	MX: 6 km E of Rayon, Chiapas.	M, E	AY859447		
R. mexicanus ROM98468	GU: 5 km E of Purulhá, Baja Verapaz.	M, E	**ON156885**		EF989911
R. mexicanus ROM98467	GU: 5 km E of Purulhá, Baja Verapaz.	M, E	AY859451	MW117094	
R. mexicanus ROM98469	GU: 5 km E of Purulhá, Baja Verapaz.	M, E	**ON156886**		
R. mexicanus ROM99880	GU: 2 km N of San Lorenzo, Sierra De Las Minas, Zacapa.	M, E	**ON156891**	**ON156957**	
R. mexicanus ROM99875	GU: 2 km N of San Lorenzo, Sierra De Las Minas, Zacapa.	M, E	**AY859452**	**ON156951**	
R. mexicanus TTU104464	ECU: 1.5 km S, 3 km W Baños, Tungurahua Lahar zone.	M, E	**ON156898**	**ON156922**	
R. mexicanus TTU104482	ECU: 1.5 km S, 1 km E Baños, Runtún.	M, E	**ON156899**	**ON156919**	

R. mexicanus TTU104770	ECU: 5 km E Baños, Represa Agoyán.	M, E	**ON156900**	**ON156920**	
R. mexicanus TTU104771	ECU: 5 km E Baños, Represa Agoyán.	M, E	**ON156904**	**ON156923**	
R. mexicanus TTU104475	ECU: 1.5 km S, 3 km W Baños, Tungurahua Lahar zone.	M, E	**ON156905**	**ON156921**	
R. mexicanus TTU104484	ECU: 1.5 km S, 1 km E Baños, Runtún.	M, E	**ON156906**	**ON156924**	
R. mexicanus UMMZ118145	GU: Aguacate river, Hacienda El Injerto, Municipio La Libertad, Huehuetenango.	GM, E			
R. mexicanus UMMZ118146	GU: Aguacate river, Hacienda El Injerto, Municipio La Libertad, Huehuetenango. .	GM, E			
R. mexicanus UMMZ118147	GU: Barillas, Hacienda Santa Gregoria, Huehuetenango.	GM, E			
R. mexicanus UMMZ118148	GU: Barillas, Hacienda Santa Gregoria, Huehuetenango.	GM, E			
R. mexicanus UMMZ118149	GU: Barillas, Hacienda Santa Gregoria, Huehuetenango.	GM, E			
R. mexicanus UMMZ118150	GU: Barillas, Hacienda Santa Gregoria, Huehuetenango.	GM, E			
R. mexicanus UMMZ118152	GU: Barillas, Hacienda Santa Gregoria, Huehuetenango.	GM, E			
R. mexicanus UMMZ118153	GU: Barillas, Hacienda Santa Gregoria, Huehuetenango.	GM, E			
R. mexicanus UMMZ118154	GU: Finca Concepción, Tucurú, Alta Verapaz.	GM, E			
R. mexicanus UMMZ118155	GU: Finca Concepción, Tucurú, Alta Verapaz.	GM, E			
R. mexicanus UMMZ127159	ECU: 8.9 km by road W Papallacta, Napo.	E			
R. mexicanus USNM570453	GU: 1.1 km NE (by road) Yalambojoch, Huehuetenango.	M, E	**ON156882**	**ON156965**	
R. mexicanus USNM570095	GU: 9.5 km NW Gualan, El Limo, Sierra de las Minas, Zacapa.	M, E	**ON156884**	**ON156954**	
R. mexicanus USNM570102	GU: 3 km S Tucuru, Finca Concepción, Tucurú, Alta Verapaz.	M, E	**ON156887**	**ON156956**	
R. mexicanus USNM569890	GU: Chelemhá Cloud Forest Reserve, Yalijux Mountain, Alta Verapaz.	M, E	**ON156888**	**ON156955**	
R. mexicanus USNM570133	GU: 9 km S of Pasmola, between km 166 and 167 on CA-14, Hotel Country Delights, Baja Verapaz.	M, E	**ON156889**		
R. mexicanus USNM570072	GU: 9.5 km NW Gualan, El Limo, Sierra de las Minas, Zacapa.	M, E	**ON156890**	**ON156953**	

R. mexicanus USNM569864	GU: Chelemhá Cloud Forest Reserve, Yalijux Mountain, Alta Verapaz.	M, E	**ON156892**	**ON156958**	
R. mexicanus USNM569952	GU: Chelemhá Cloud Forest Reserve, Yalijux Mountain, Alta Verapaz.	M, E	**ON156893**	**ON156959**	
R. mexicanus USNM570436	GU: 3.5 km N (by air) Aldea la Trinidad, Huehuetenango.	M, E	**ON156894**	**ON156966**	
R. mexicanus USNM570446	GU: 1.7 km NE (by road) Yalambojoch, Huehuetenango.	M, E	**ON156895**	**ON156967**	
R. mexicanus UV2213	MX: 3 km N Zongolica, Municipio Zongolica, Veracruz.	M, GM, E	**ON156866**		
R. brevirostris ROM97308	CR: Monte Verde, Puntarenas.	M	EF990005		EF989906
R. brevirostris ROM116845	CR: Monte Verde, Puntarenas.	M	EF990007		EF989908
R. brevirostris ROM116841	CR: foothills near Pico Blanco, San José, San Antonio de Escazú.	M	EF990011		EF989912
R. brevirostris ROM116839	NIC: Cerro Madera, Ometepe Island.	M	EF990016		EF989917
R. brevirostris ROM116804	CR: 10 km E of Sucre, Juan Castro Blanco National Park, Alajuela.	M	EF990017		EF989918
R. brevirostris ROM116824	NIC: Cerro Madera, Ometepe Island.	M	EF990020		EF989921
R. brevirostris MVZ174401	CR: Colima Tapanti, 1.6 km S Tapanti Bridge over Rio Grande de Orosi, Cartago.	M	AF108709		
R. darienenis ROM116311	PAN: Mount Pirri, Darien Province.	M	EF990015		EF989916
R. garichensis MSB262794	PAN: Jurufungo, Chiriqui, Renacimiento, La Amistad International Park.	M	**ON156907**	**ON156918**	
R. sp. Cartago ROM116835	CR: Cerro de la Carpentera, Iztaru Scout Camp, Cartago.	M	EF990012		EF989913
R. sp. Poas ROM114291	CR: Volcan Poas National Park, Alajuela.	M	EF990018		EF989919
R. sp. Poas ROM116805	CR: Volcan Poas National Park, Alajuela.	M	EF990019		EF989920
R. gracilis TTU34814	ES: about 3 miles NW San Luis Talpa, La Paz.	M	**ON156908**	**ON156916**	
R. gracilis TTU34820	ES: about 3 miles NW San Luis Talpa, La Paz.	M	**ON156909**	**ON156917**	
R. gracilis ROM95890	MX: 52 km SW of Champoton, Municipio Champoton, Campeche.	M	AY859432		EF989905
R. gracilis FN30426	MX: Laguna Becanchen, Yucatán.	M	AY293817		
R. gracilis ASNHC6370	MX: Laguna Becanchen, Yucatán.	M	AY859431		

R. spectabilis ASNHC2140	MX: 30 km SE San Miguel, Isla Cozumel, Quintana Roo.	M	AY859462		
R. spectabilis ROM97733	MX: 1.5 km N of El Cedral, Isla Cozumel, Quintana Roo.	M	EF990022		EF989923
R. spectabilis ASNHC2139	MX: 30 km SE San Miguel, Isla Cozumel, Quintana Roo.	M	EF990021		EF989922
R. microdon Ecosur1930	MX: Cerro Mozotal 30 km N Motozintla by road Buenos Aires-El Porvenir, Municipio El Porvenir, Chiapas.	M	MW117046	MW117100	**ON156969**
R. microdon Ecosur2671	MX: 2 km NE San Cristóbal de las Casas, Reserva Ecológica Huitepec Hillside, Chiapas.	M	MW117051	MW117101	
R. microdon ROM98300	GU: 12 km NW of Santa Eulalia (by road), Huehuetenango.	M	EF990014		EF989914
R. microdon ROM98382	GU: 16 km NW of Santa Eulalia (by road), Huehuetenango.	M	AY859458		EF989915
R. tenuirostris BYU 14479	MX: Cerro Tzontehuitz, 13 km NE San Cristóbal de las Casas, Municipio Chamula, Chiapas.	M	AY859463	MW117095	
R. tenuirostris ECOSUR1817	MX: Cerro Tzontehuitz, 11 km NE San Cristóbal de las Casas, Municipio Chamula, Chiapas.	M	MW117045	MW117096	
R. cherrii LSUMNS25169	CR: 1 km (by road) SW Poas, San José.	M	MW117038	MW117074	
R. cherrii LSUMNS381	CR: Cartago.	M	**ON156912**	**ON156913**	
R. cherrii LSUMNS466	CR: San José.	M	**ON156911**	**ON156914**	
R. cherrii LSUMNS380	CR: Cartago.	M	MW117037	MW117077	
R. cherrii MSB61867	CR: 2 km NE Getzemani, Heredia.	M	**ON156910**	**ON156915**	
R. megalotis ASNHC2133	MX: 3 km S of Parres, Ciudad de México.	M	EF990008		EF989909
R. megalotis ASNHC2136	MX: 3 km S of Parres, Ciudad de México.	M	EF990009		EF989910
R. megalotis CMC1072	MX: 4.7 km NE Teziutlán (by road), Municipio Teziutlán, Puebla.	M	HQ269732	HQ269795	
R. sumichrasti ROM98383	GU: 10 km SW of Santa Eulalia, Santa Eulalia, Huehuetenango.	M	HQ269715	HQ269787	EF989924
R. sumichrasti ROM98384	GU: 15 km SW of Santa Apolonia, Santa Apolonia, Chimaltenango.	M	HQ269716	HQ269788	EF989925
R. fulvescens BYU20914	MX: Río de la Arena 6 km E (by road) Pinotepa Nacional, Municipio Pinotepa Nacional.	M	HQ269730	HQ269794	
R. fulvescens ASNHC3465	MX: 4.8 km N of Santiago, Santiago, Nayarit.	M	EF990000		EF989901
