# Supplementary material for: Species delimitation and integrative taxonomy of the Reithrodontomys mexicanus (Rodentia: Cricetidae) cryptic complex
Source: Ecol Evol. 2023 Jul 30;13(8):e10355. doi: 10.1002/ece3.10355 (PMC10387591; doi:10.1002/ece3.10355)
Supplement: Supplementary file 2 — Appendix S2. [file ECE3-13-e10355-s005.pdf]

Appendix 2

Configurations of landmark (black points) and semi-landmark (blue points) digitized in the ventral and dorsal views of the skull. Landmark names and anatomical position are defined at the bottom of the figure. Reference specimen: An adult male of *Reithrodontomys mexicanus* deposited at Monte L. Bean Life Science Museum, Brigham Young University (BYU 15439).

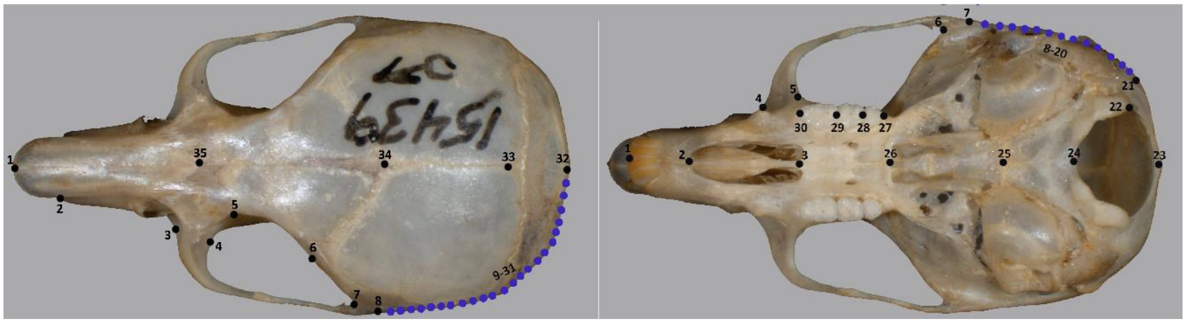

	Dorsal view		Ventral view
1	Rostralmost point of the nasal bone.	1	Rostralmost point of the upper incisor tooth next to the midline.
2	Anteriormost point of suture between nasal bone and nasal process of the incisive.	2	Anteriormost point of the incisive foramen.
3	Rostral end of zygomatic plate in a dorsal projection.	3	Posteriormost point of the incisive foramen.
4	Anteriormost point of the orbit in a dorsal projection.	4	Rostral end of zygomatic plate in a ventral projection.
5	Narrowest point of the interorbital region.	5	Anteriormost point of the orbit in a ventral projection.
6	Rostralmost point of the parietal bone.	6	Caudalmost point of the orbit in a ventral projection.
7	Caudalmost point of the orbit in a dorsal projection.	7	Posterior end of zygomatic bar in a ventral projection.
8	Posterior end of zygomatic bar in a dorsal projection.	8-20	Semi-landmarks
		21	Lateral margin of the basioccipital.
9-31	Semi-landmarks	22	Lateral margin of the foramen magnum.
32	Caudal end of the curvature of the occipital bone.	23	Posteriormost point of the occipital foramen in the midline.
33	Intersection of the sagittal and parietal-interparietal sutures.	24	Anteriormost point of the occipital foramen in the midline.
34	Intersection of the coronal and sagittal sutures.	25	Midpoint of suture between basisphenoid and basioccipital.
35	Intersection of the naso-frontal suture in the midline.	26	Posteriormost extent of palate at the midline.
		27	Posteriormost point of the third molar.
		28	Contact point between second and third molars.
		29	Contact point between first and second molars.
		30	Anteriormost point of the first molar.
