# Supplementary material for: Species delimitation and integrative taxonomy of the Reithrodontomys mexicanus (Rodentia: Cricetidae) cryptic complex
Source: Ecol Evol. 2023 Jul 30;13(8):e10355. doi: 10.1002/ece3.10355 (PMC10387591; doi:10.1002/ece3.10355)
Supplement: Supplementary file 3 — Appendix S3. [file ECE3-13-e10355-s007.pdf]

Appendix 2

Tables 1-4. Matrixes of Kimura 2-parameter genetic distances (%) for cytochrome-b gene sequence data between the *Reithrodontomys mexicanus* species group taxa. Taxon labels correspond to species delimited by mPTP, bGMYC, and STACEY methods.

Species delimitation (mPTP)

	1	2	3	4	5	6	7	8	9	10	11	12	13	14
R. mexicanus clade I														
R. mexicanus clade IIA	15.92													
R. mexicanus clade IIB	15.92	4.96												
R. mexicanus clade IIIA	16.86	8.26	8.72											
R. mexicanus clade IIIB1	13.20	5.20	7.20	1.60										
R. mexicanus clade IIIB2	14.30	6.50	8.60	3.60	2.20									
R. mexicanus clade IIIB3	13.50	4.30	6.10	3.10	1.50	3.20								
R. mexicanus clade IIIB4	15.70	7.10	9.0	3.40	2.20	4.20	3.90							
R. brevirostris clade IA	14.40	3.20	4.10	5.70	5.20	6.50	4.80	8.10						
R. brevirostris clade IB	15.60	4.80	5.10	6.70	6.90	8.70	6.50	9.50	2.80					
R. darienensis	16.68	8.48	7.69	5.60	5.0	7.60	5.80	8.30	8.30	9.70				
R. garichensis	18.18	14.59	12.92	13.60	12.80	14.70	12.90	13.30	12.60	13.20	15.62			
R. sp. Volcan Poas	17.12	13.83	13.98	14.0	12.30	12.90	12.40	14.20	12.50	13.60	13.95	11.31		
R. gracilis + R. spectabilis	14.70	13.43	13.26	12.0	14.10	15.30	12.50	14.0	11.60	12.90	13.86	16.60	15.23	
R. gracilis El Salvador	15.16	13.43	14.50	15.30	15.70	16.30	14.0	15.80	13.30	14.20	15.55	17.38	16.38	7.91

Species delimitation (bGMYC)

	1	2	3	4	5	6	7	8	9	10
R. mexicanus clade I										
R. mexicanus clade IIA	15.92									
R. mexicanus clade IIB	15.92	4.96								
R. mexicanus clade IIIA	16.86	8.26	8.72							
R. mexicanus clade IIIB	15.88	8.32	8.96	6.06						
R. brevirostris	16.15	5.68	5.23	8.31	8.34					
R. darienensis	16.68	8.48	7.69	8.30	7.54	9.22				
R. garichensis	18.18	14.59	12.92	16.09	14.24	14.0	15.62			
R. sp. Volcan Poas	17.12	13.83	13.98	14.86	13.93	13.37	13.95	11.31		
R. gracilis + R. spectabilis	14.70	13.43	13.26	15.53	14.56	12.91	13.86	16.60	15.23	
R. gracilis El Salvador	15.16	13.43	14.50	16.72	16.22	14.29	15.55	17.38	16.38	7.91

Species delimitation (STACEY: cytb + Fgb- I7)*

	1	2	3	4	5	6	7	8	9
R. mexicanus clade I									
R. mexicanus clade IIA1	14.60								
R. mexicanus clade IIA2	13.30	2.0							
R. mexicanus clade IIA3	14.80	2.40	2.70						
R. mexicanus clade IIB	15.92	3.90	4.0	4.40					
R. mexicanus clade IIIA	16.86	4.90	5.0	5.20	8.72				
R. mexicanus clade IIIB1	12.60	5.80	5.30	5.70	7.10	1.50			
R. mexicanus clade IIIB2	15.70	7.60	8.50	8.10	9.0	3.90	2.10		
R. garichensis	18.18	13.40	12.70	13.20	12.92	16.09	12.50	13.30	
R. gracilis El Salvador	15.16	12.60	12.60	12.30	14.50	16.72	15.0	15.80	17.38

* Concatenated data set not available for *R. brevirostris*, *R. darienensis*, *R. sp*. Volcan Poas, and *R. gracilis* + *R. spectabilis*.

Species delimitation (STACEY: cytb + IRBP)*

	1	2	3	4	5	6	7	8	9
R. mexicanus clade I									
R. mexicanus clade IIA1	15.92								
R. mexicanus clade IIA2	14.80	2.0							
R. brevirostris clade IA	14.70	3.50	4.80						
R. brevirostris clade IB	15.10	4.0	4.90	1.30					
R. brevirostris clade IC	15.60	4.70	6.30	2.80	3.60				
R. darienensis	16.68	7.30	7.80	8.60	8.80	9.70			
R. garichensis	18.18	12.70	13.20	12.80	13.40	13.20	15.62		
R. sp. Volcan Poas	17.12	12.60	13.20	12.80	13.10	13.60	13.95	11.31	
R. gracilis + *R. spectabilis*	14.70	11.50	12.70	11.70	12.0	12.80	13.86	16.60	15.23

* Concatenated data set not available for *R. mexicanus* clade IIB, *R. mexicanus* clade III (Colombia and Ecuador), and *R. gracilis* El Salvador.
