# Supplementary material for: Species delimitation and integrative taxonomy of the Reithrodontomys mexicanus (Rodentia: Cricetidae) cryptic complex
Source: Ecol Evol. 2023 Jul 30;13(8):e10355. doi: 10.1002/ece3.10355 (PMC10387591; doi:10.1002/ece3.10355)
Supplement: Supplementary file 4 — Appendix S4. [file ECE3-13-e10355-s004.pdf]

Appendix 3

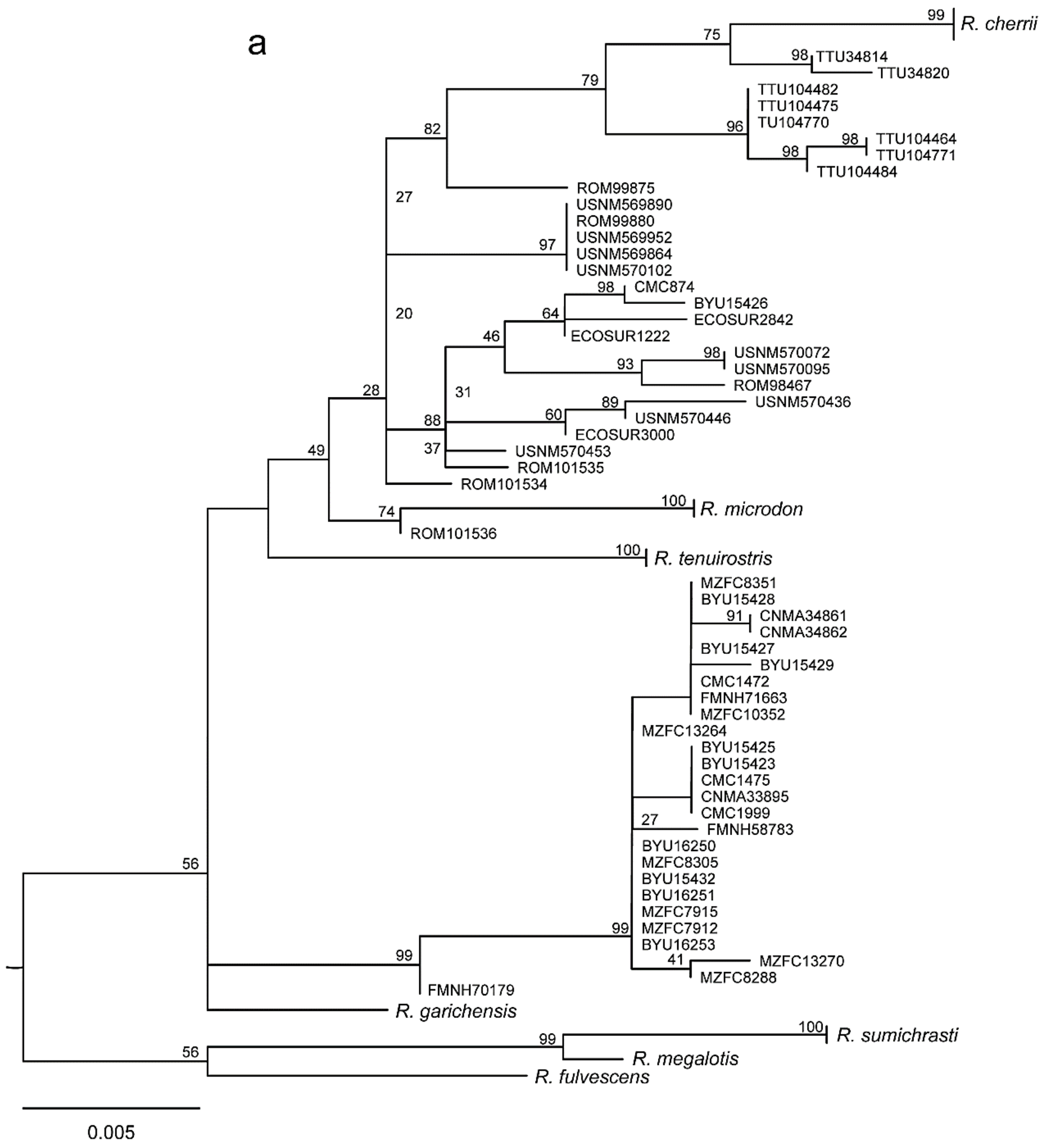

a
99
R. cherrii
75
98
TTU34814
TTU34820
79
TTU104482
TTU104475
TU104770
96
98
TTU104464
TTU104771
98
TTU104484
82
ROM99875
27
USNM569890
ROM99880
USNM569952
USNM569864
USNM570102
97
98
CMC874
BYU15426
64
ECOSUR2842
ECOSUR1222
46
20
98
USNM570072
USNM570095
93
ROM98467
31
89
USNM570436
60
USNM570446
ECOSUR3000
28
88
37
USNM570453
ROM101535
49
ROM101534
74
100
R. microdon
ROM101536
100
R. tenuirostris
MZFC8351
BYU15428
91
CNMA34861
CNMA34862
BYU15427
BYU15429
CMC1472
FMNH71663
MZFC10352
MZFC13264
BYU15425
BYU15423
CMC1475
CNMA33895
CMC1999
27
FMNH58783
BYU16250
MZFC8305
BYU15432
BYU16251
MZFC7915
56
99
MZFC7912
BYU16253
41
MZFC13270
MZFC8288
99
FMNH70179
R. garichensis
99
100
R. sumichrasti
56
R. megalotis
R. fulvescens
0.005

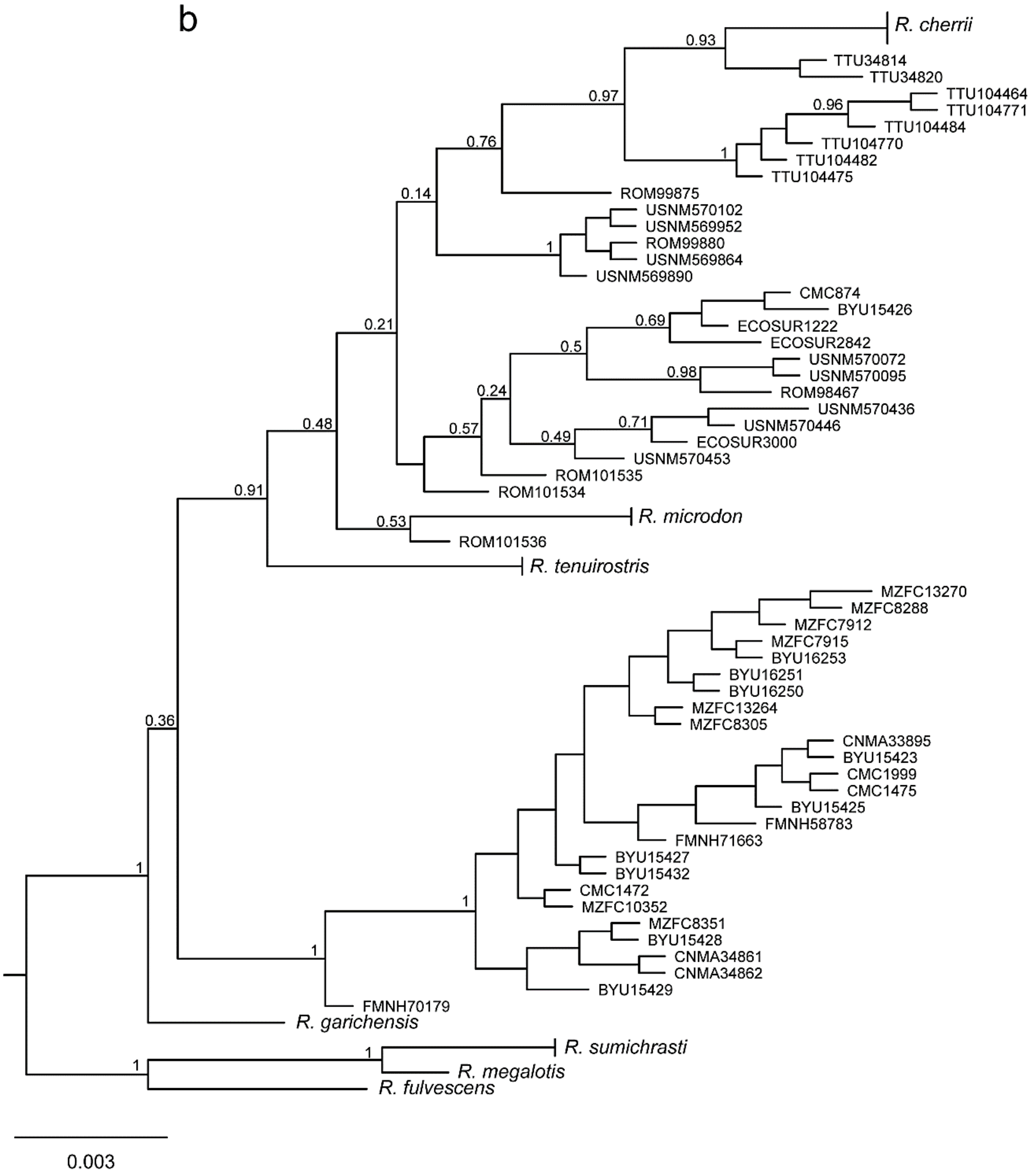

Phylogenetic relationships among species of the *Reithrodontomys mexicanus* group (Rodentia: Cricetidae) using the intron 7 of the beta fibrinogen sequences data set and the reconstructive methods of Maximum Likelihood (a) and Bayesian Inference (b). Values on branches represent nodal support. Terminal labels correspond to mammal collection voucher numbers (see Appendix 1).
