# Supplementary material for: Species delimitation and integrative taxonomy of the Reithrodontomys mexicanus (Rodentia: Cricetidae) cryptic complex
Source: Ecol Evol. 2023 Jul 30;13(8):e10355. doi: 10.1002/ece3.10355 (PMC10387591; doi:10.1002/ece3.10355)
Supplement: Supplementary file 5 — Appendix S5. [file ECE3-13-e10355-s008.pdf]

Appendix 4

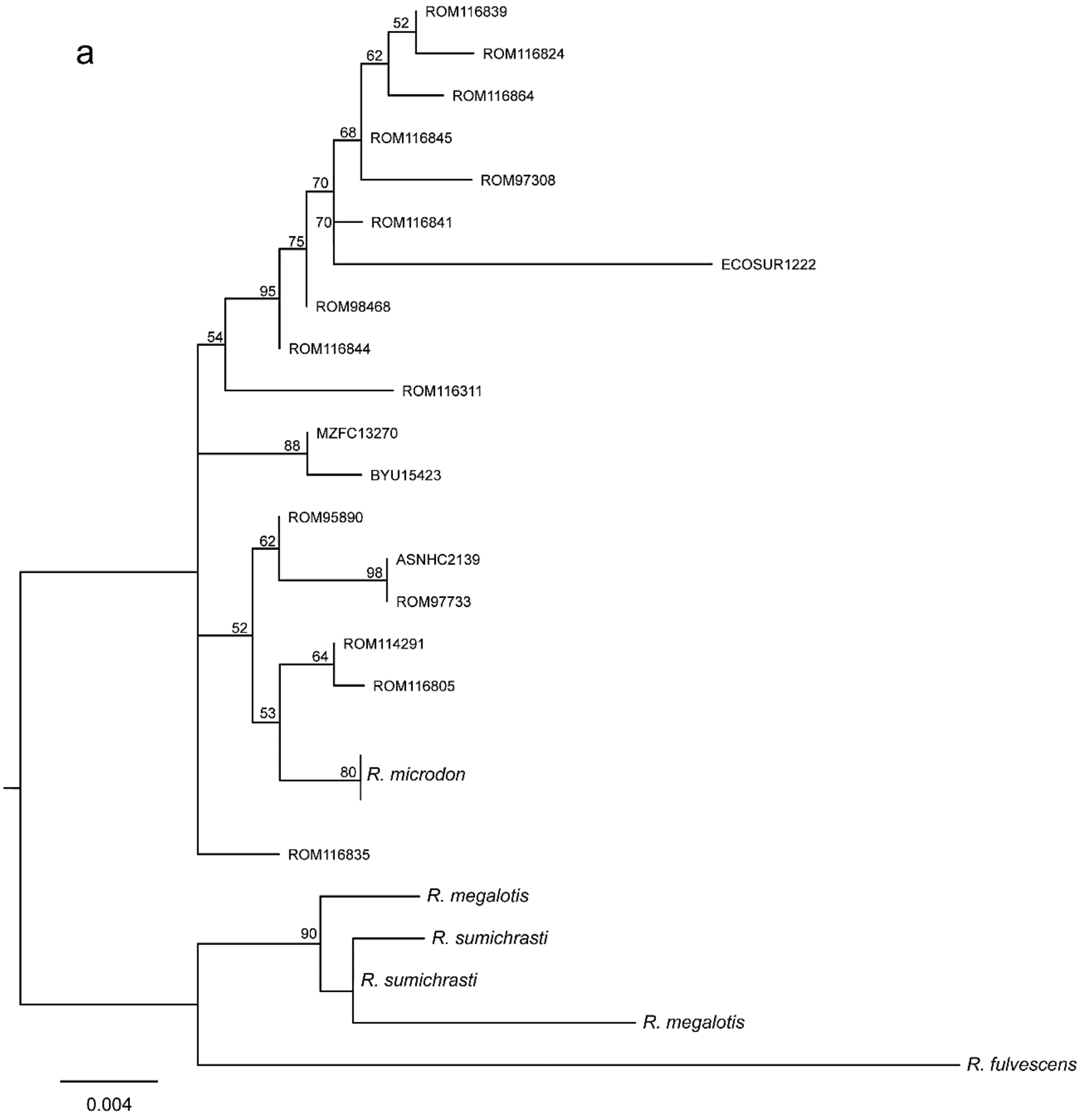
a
ROM116839
ROM116824
ROM116864
ROM116845
ROM97308
ROM116841
ECOSUR1222
ROM98468
ROM116844
ROM116311
MZFC13270
BYU15423
ROM95890
ASNHC2139
ROM97733
ROM114291
ROM116805
R. microdon
ROM116835
R. megalotis
R. sumichrasti
R. sumichrasti
R. megalotis
R. fulvescens
52
62
68
70
70
75
95
54
88
62
98
52
64
53
80
90
0.004

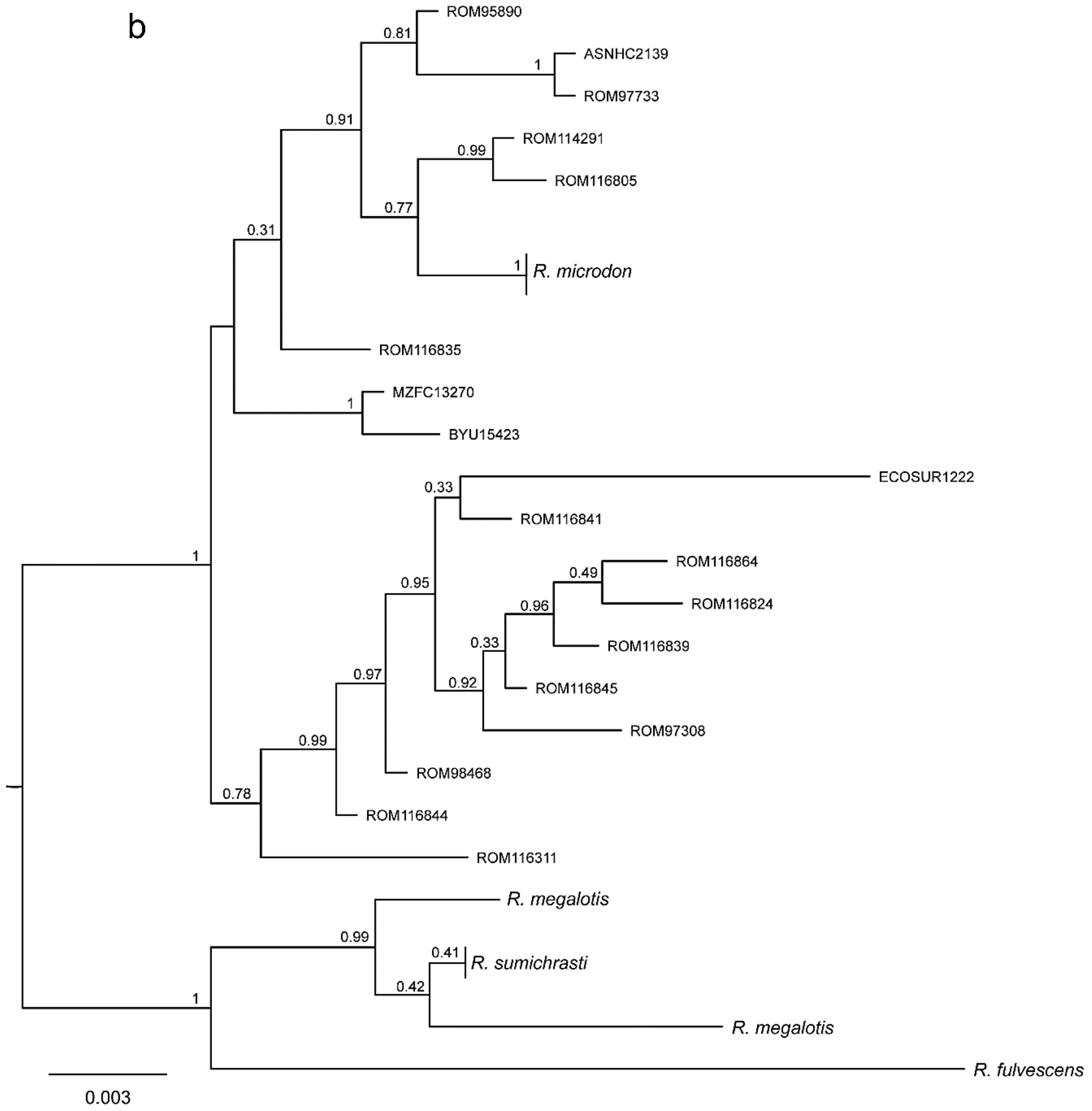

Phylogenetic relationships among species of the *Reithrodontomys mexicanus* group (Rodentia: Cricetidae) using the Interphotoreceptor retinoid-binding protein sequences data set and the reconstructive methods of Maximum Likelihood (a) and Bayesian Inference (b). Values on branches represent nodal support. Terminal labels correspond to mammal collection voucher numbers (see Appendix 1).
