# Supplementary material for: Species delimitation and integrative taxonomy of the Reithrodontomys mexicanus (Rodentia: Cricetidae) cryptic complex
Source: Ecol Evol. 2023 Jul 30;13(8):e10355. doi: 10.1002/ece3.10355 (PMC10387591; doi:10.1002/ece3.10355)
Supplement: Supplementary file 6 — Appendix S6. [file ECE3-13-e10355-s002.pdf]

Appendix 5

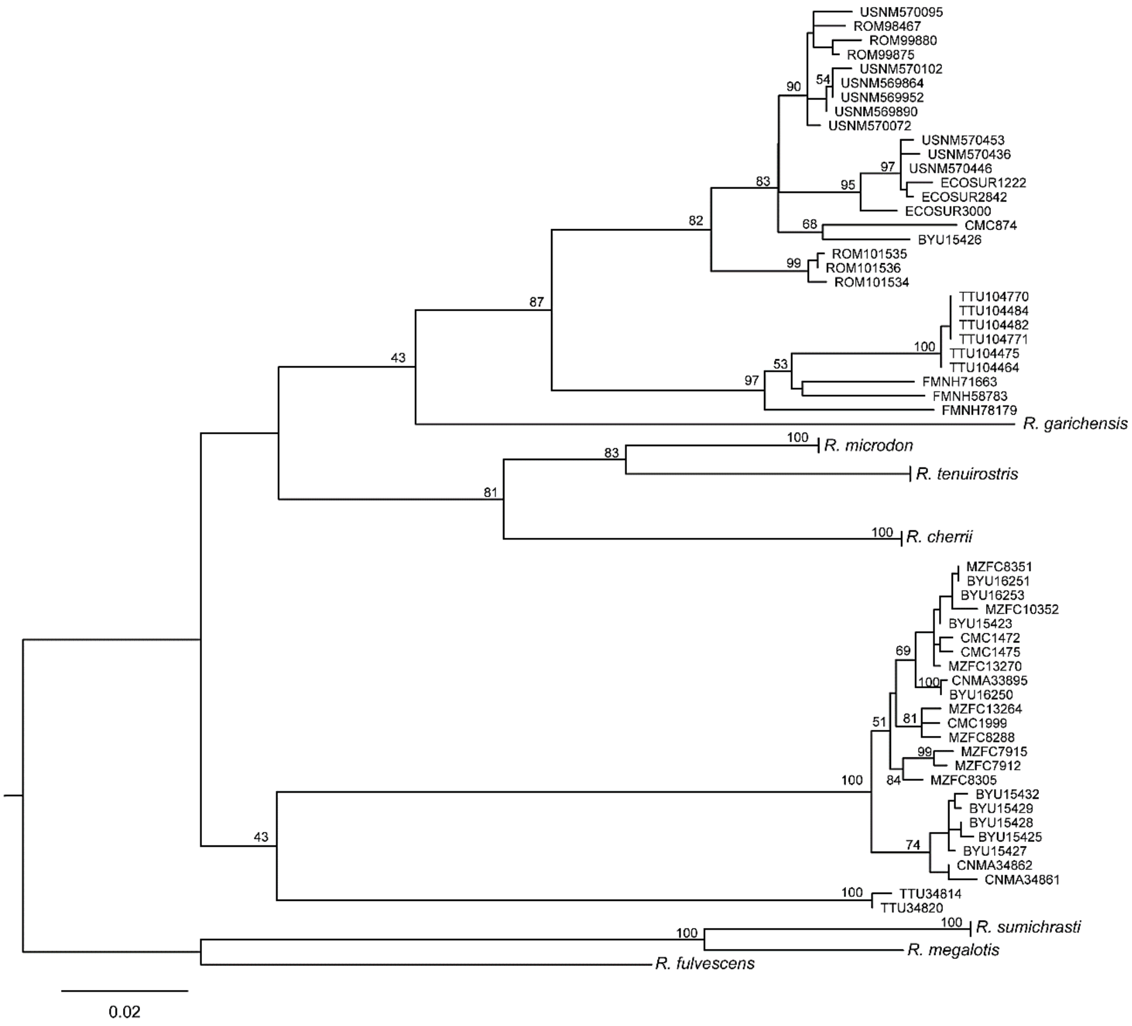

Phylogenetic relationships among species of the *Reithrodontomys mexicanus* group (Rodentia: Cricetidae) using sequence data of the concatenated dataset Cytochrome b + Intron 7 of the beta fibrinogen and the reconstructive method of Maximum Likelihood. Values on branches represent nodal support. Terminal labels correspond to mammal collection voucher numbers (see Appendix 1).
