# Supplementary material for: Species delimitation and integrative taxonomy of the Reithrodontomys mexicanus (Rodentia: Cricetidae) cryptic complex
Source: Ecol Evol. 2023 Jul 30;13(8):e10355. doi: 10.1002/ece3.10355 (PMC10387591; doi:10.1002/ece3.10355)

Appendix 7

Partial-ROC values and distribution of AUC ratios; red distribution represents the null model; blue distribution represents the distribution of expectations using a random points percentage of 50% of the total available points and 1000 resampling replicates.

Reithrodontomys mexicanus clade I

Mean AUC ratio after 1000 simulations: 1.799753 ($p = 0$)

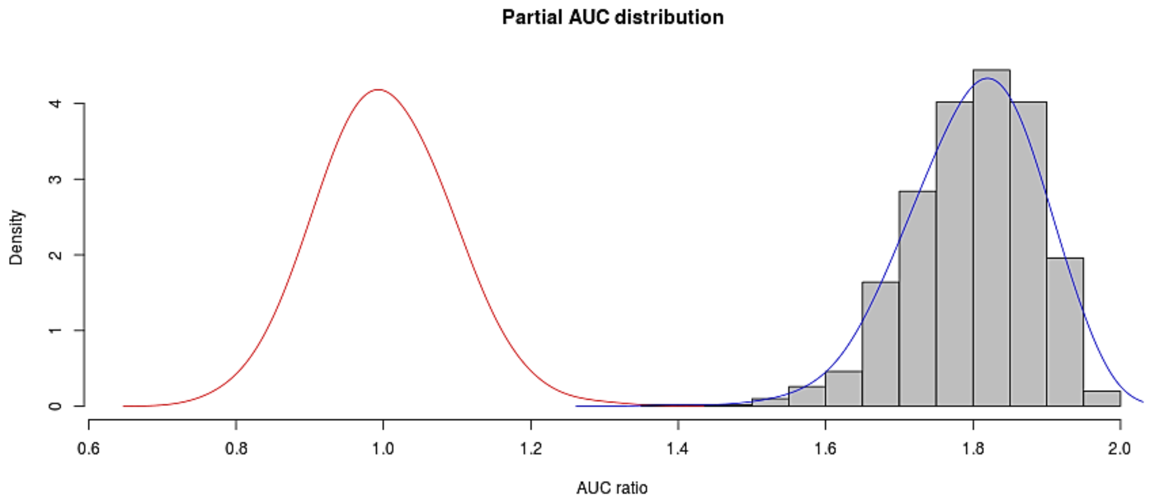

Reithrodontomys mexicanus clade IIA

Mean AUC ratio after 1000 simulations: 1.814829 ($p = 0$)

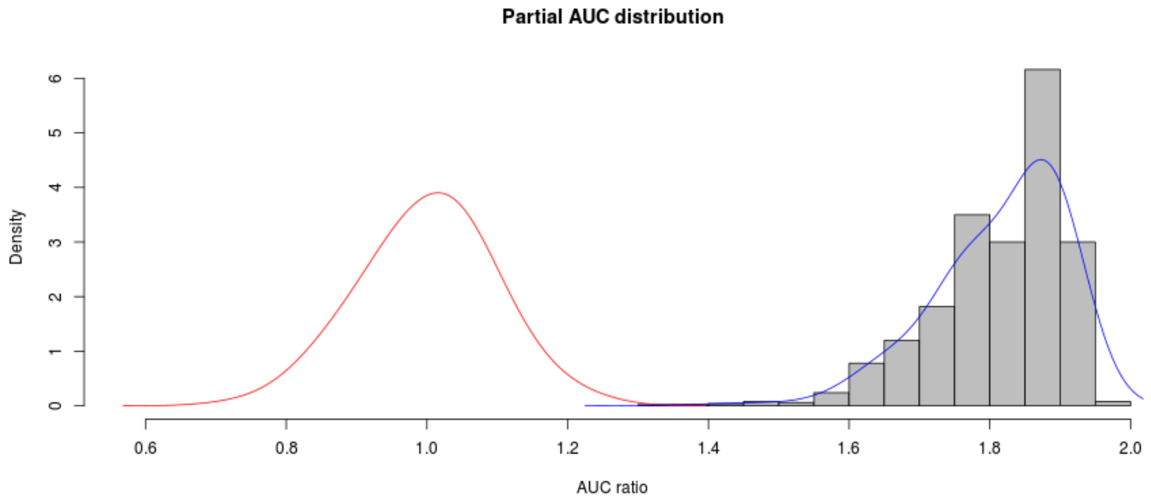

Reithrodontomys mexicanus clade IIIA

Mean AUC ratio after 1000 simulations: 1.403082 (p = 0.021)

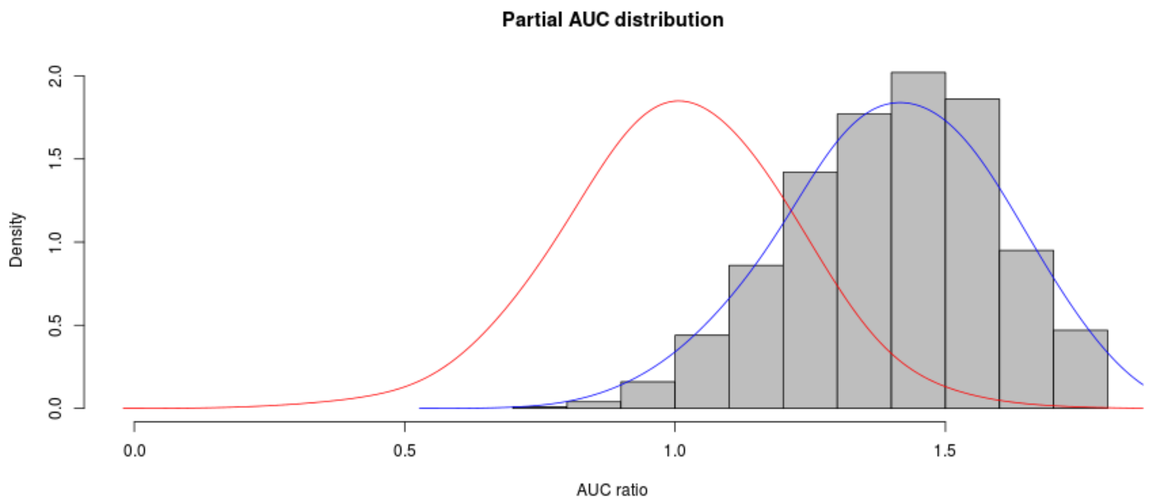

Reithrodontomys mexicanus clade IIIB

Mean AUC ratio after 1000 simulations: 1.777717 (p = 0)

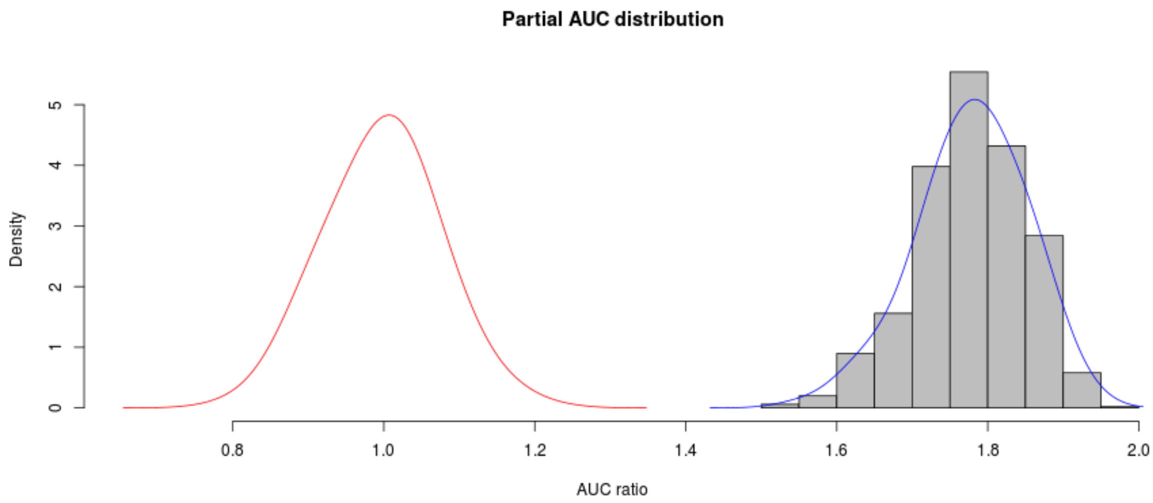

Supplement: Supplementary file 8 — Appendix S8. [file ECE3-13-e10355-s001.pdf]
